# A mixed methods evaluation of family-driven care implementation in juvenile justice agencies in Georgia

**DOI:** 10.1186/s40352-024-00261-0

**Published:** 2024-02-26

**Authors:** Kaitlin N. Piper, Alexandra Jahn, Cam Escoffery, Briana Woods-Jaeger, Amy Nunn, David P. Schwartz, Cathy Smith-Curry, Jessica Sales

**Affiliations:** 1 Department of Behavioral, Social, and Health Education Sciences, Rollins School of Public Health, Emory University, 1518 Clifton Road, Atlanta, GA USA; 2 Department of Social and Behavioral Sciences, Brown University School of Public Health, Providence, RI USA; 3 Department of Juvenile Justice, Atlanta, GA USA

**Keywords:** Juvenile justice, Family-driven care, Family engagement, Implementation science, Behavioral health services

## Abstract

**Background:**

Improving family engagement in juvenile justice (JJ) system behavioral health services is a high priority for JJ systems, reform organizations, and family advocacy groups across the United States. Family-driven care (FDC) is a family engagement framework used by youth-serving systems to elevate family voice and decision-making power at all levels of the organization. Key domains of a family-driven system of care include: 1) identifying and involving families in all processes, 2) informing families with accurate, understandable, and transparent information, 3) collaborating with families to make decisions and plan treatments, 4) responding to family diversity and inclusion, 5) partnering with families to make organizational decisions and policy changes, 6) providing opportunities for family peer support, 7) providing logistical support to help families overcome barriers to participation, and 8) addressing family health and functioning. FDC enhances family participation, empowerment, and decision-making power in youth services; ultimately, improving youth and family behavioral health outcomes, enhancing family-child connectedness, and reducing youth recidivism in the JJ setting.

**Methods:**

We evaluated staff-perceived adoption of the eight domains of FDC across detention and community services agencies in the state of Georgia. We collected mixed methods data involving surveys and in-depth qualitative interviews with JJ system administrators, staff, and practitioners between November 2021- July 2022. In total, 140 individuals from 61 unique JJ agencies participated in surveys; and 16 JJ key informants participated in qualitative interviews.

**Results:**

FDC domains with the highest perceived adoption across agencies included identifying and involving families, informing families, collaborative decision-making and treatment planning, and family diversity and inclusion. Other domains that had mixed or lower perceived adoption included involving families in organizational feedback and policy making, family peer support, logistical support, and family health and functioning. Adoption of FDC domains differed across staff and organizational characteristics.

**Conclusions:**

Findings from this mixed methods assessment will inform strategic planning for the scale-up of FDC strategies across JJ agencies in the state, and serve as a template for assessing strengths and weaknesses in the application of family engagement practices in systems nationally.

**Supplementary Information:**

The online version contains supplementary material available at 10.1186/s40352-024-00261-0.

## Introduction

In 2019, approximately 700,000 youth under the age of 18 were arrested by law enforcement in the United States (Puzzanchera, 2021). Among these justice-involved youth, studies estimate that between 50–70% meet criteria for at least one psychiatric disorder (Burke et al., 2015; Teplin et al., 2002; Wasserman et al., 2010), compared to about 19% in the general adolescent population (McCance-Katz, 2019; Merikangas et al., 2010). Specifically, studies estimate that approximately 34% of justice-involved youth have a substance use disorder, 27% have a disruptive behavior disorder, 20% have an anxiety disorder, 14% have attempted suicide in their lifetime, and 8% have an affective disorder (Wasserman et al., 2010). Among youth in the juvenile justice (JJ) system, behavioral health disorders are one of the most reliable predictors of recidivism (Hoeve et al., 2014; Machteld Hoeve et al., 2013; Schubert et al., 2011; Van der Put et al., 2014), and they can lead to lifelong health and wellbeing complications such as suicidal ideation (Nolen et al., 2008; Wasserman & McReynolds, 2006), trauma exposure (Wasserman et al., 2010), elevated sexual risk behaviors (Elkington et al., 2010; Teplin et al., 2003), and reductions in academic achievement (Arthur et al., 2015). Despite the disproportionate burden of substance use and mental health disorders among this population, very few youth in the JJ system actually receive treatment. It is estimated that only 20% of justice-involved youth needing mental health care actually initiate treatment, and less than 10% in need of substance use service initiate treatment (Burke et al., 2015; Wasserman et al., 2021).

Families play a critical role in improving the need-treatment gap among justice-involved youth. Family engagement in youth’s behavioral health care (e.g., including families in services and decisions related to the care of their child) is linked to increased treatment initation and sustainment, as well as impoved treatment outcomes among justice-involve youth (Haine-Schlagel & Walsh, 2015; Henggeler et al., 2002; Hornberger & Smith, 2011; Liddle et al., 2011; Lindsey et al., 2013). Youth with supportive families also are less likely to reoffend and become further involved in the system (Latimer, 2001). There are many explanations for the strong relationship between family involvement and the wellbeing of justice-involved youth. For instance, in the JJ setting, families have numerous roles including helping their child navigate the system, providing emotional support during a particularly stressful period, reinforcing positive behaviors or treatment plans, and providing tangible support (e.g., scheduling and transporting youth to treatment appointments) (Osher et al., 2008a; b; Paik, 2017). In addition, families can provide valuable information about the child’s background, culture, and behavioral health needs that can lead to more tailored and effective interventions and treatment plans (Hornberger & Smith, 2011). Additionally, when families are involved in the treatment process, it can improve family connectedness, promote healthy behaviors among the family unit, and address issues that may have contributed to youth’s involvement in the system (Liddle et al., 2009). Due to the importance of families, the National Institutes on Drug Abuse (NIDA), the American Academy of Child and Adolescent Psychiatry (AACAP), and other expert panels agree that family involvement is a core component of adolescent behavioral health treatment (AACAP, 2003; National Federation of Families for Childrens Mental Health, 2008; National Institute on Drug Abuse, 2014). In addition to the evidence supporting family engagement in JJ settings, high-level calls-to-action from JJ reform organizations and family-advocacy groups have also highlighted the critical need for family participation JJ systems (Arya, 2013; Burke, Mulvey, Schubert, & Garbin, 2014a; Justice for Families, 2012a, 2012b; OJJDP, 2013; Paik, 2017; Pennell et al., 2011; Shanahan & diZerega, 2016; Vera Institute of Justice, 2014).

In response to this evidence and calls-to-action, family engagement in service delivery is currently one of the top priority areas for JJ organizations nationally (Office of Juvenile Justice & Delinquency Prevention, 2010). Over the past decade, there has been a paradigm shift in JJ systems, where systems are now addressing the underlying causes of delinquency (through reentry and aftercare services, probation, and diversion programs) rather than focusing on punitive incarceration. In fact, between the years 2000 and 2017, the number of youth placed in locked facilities has decreased by 60%, with the majority of youth now receiving at-home placements within their families and communities (Prison Policy Initiative, 2019). Instead of focusing on restrictive confinement, JJ systems are working to improve youth long-term success by strengthening their support systems and improving their involvement in services within their communities, which relies heavily on the engagement of parents and families (Nellis, Wayman, & Schirmer, 2009; (Prison Policy Initiative, 2019). Despite prioritizing youth and family wellbeing, JJ systems have encountered numerous policy and practice barriers towards effectively collaborating with families (Amani et al., 2018; Burke et al., 2014a, b; Peterson-Badali & Broeking, 2010). A survey of justice correctional leaders identified family engagement as the most challenging issue to implement practically in their systems (Center for Juvenile Justice Reform, 2008). Specifically, JJ systems are struggling to create spaces for trusting, egalitarian relationships with families due to the punitive and coercive nature of the system (e.g., requiring compliance from youth and families), power differentials between staff and families, and a culture that historically minimized the role of families, blamed and shamed families for their child’s behavior, and excluded families from decisions (Arya, 2013; Pennell et al., 2011; Shanahan & diZerega, 2016).

Family engagement strategies, principles, and frameworks can provide guidance to mitigate these obstacles. One framework for guiding family engagement in JJ systems is Family-Driven Care (FDC), which was collaboratively developed by the Federation of Families and the Substance Abuse and Mental Health Services Administration (SAMHSA). The Federation and SAMHSA designed FDC to be flexibly applied to a variety of youth-serving organizations, and they included tailored guidance and priorities for JJ agencies (National Federation of Families for Childrens Mental Health, 2008; Spencer et al., 2010). In brief, FDC empowers families’ voices, so they have a primary decision-making role in the care of their own children as well as in the organizational policies and procedures governing care for all children in the system (Osher et al., 2008b). Elements of FDC include: 1) identifying and involving families in all processes, 2) informing families with accurate, understandable, and transparent information, 3) collaborating with families to make decisions and plan treatments, 4) responding to family diversity and inclusion, 5) partnering with families to make organizational decisions and policy changes, 6) providing opportunities for family peer support, 7) providing logistical support to help families overcome barriers to participation, and 8) addressing family wellbeing and functioning. These eight FDC domains are defined in more detail in Table 1. Evidence from child-serving systems (including pediatrics, education, child welfare, and child behavioral health settings) suggests that FDC leads to improved family and child outcomes, including increased family satisfaction and service engagement, improved family functioning, and improved child health and behavior (Dunst & Trivette, 2009a, 2009b; Dunst & Trivette, 2009a, 2009b; Dunst,Trivette, & Hamby, 2007; Dunst,Trivette, & Hamby, 2007; Geurts et al., 2012; Horwitz, Chamberlain, Landsverk, & Mullican, 2010; McWayne et al., 2004; Williamson & Gray, 2011).

**Table 1:** Family-driven care domains, definitions, and example applications

Domain	Description	Example Strategy Applications in JJ Settings
1. Identifying and Involving Family Voices	• Methods used to identify members of the family unit and other supportive adults who should be involved in JJ processes • Define family broadly to include traditional and non-traditional caregivers and other supportive adults • Ensure that a family voice is present during all JJ decisions and processes	• Juvenile Relational Inquiry Tool (JRIT) (Shanahan & Agudelo, 2012) • Family Finding (Welti, Wilkins, & Malm, 2021) • Inclusive definition of family (Arya, 2013)
2. Informing Families	• Families are given complete, accurate, and understandable information about JJ processes, so they can make informed decisions • Families are informed about resources/services and how to access them	• Family orientations (S. Walker et al., 2011) • Family handbooks and resource guides (S. Walker et al., 2011)
3. Collaborative Decision-Making and Care Planning	• Families, youth, and professionals work collaboratively to make decisions and develop treatment plans for justice-involved youth • Family’s needs and preferences are prioritized	• Family group decision-making (Annie E. Casey Foundation, 2014; Lewis & Judge, 2005; Pennsylvania’s Family Group Decision Making (FGDM) Leadership Team, 2009)
4. Family Diversity and Inclusion	• Families’ cultural backgrounds are respected throughout their involvement with the JJ system • Staff advance their cultural and linguistic responsiveness	• Provision of culturally appropriate services (DMC Action Network, 2009) • Cultural sensitivity training for staff (Hoytt et al., 2002; Willison, 2010) • Linguistic Competency (Hoytt et al., 2002)
5. Organizational Feedback and Decision-Making	• Families provide feedback to the JJ system • Families are involved in policy-making and practice decisions for the JJ system	• Family advisory boards or committees (Arya, 2013)
6. Family Peer Support	• Families engage in peer support activities to reduce isolation, gather and disseminate accurate information, and strengthen the family voice	• Family peer specialists (e.g., peer navigators, peer advocates, peer educators) (S. Walker et al., 2011) • Family support groups (Cataldo & Ford, 2010)
7. Logistical Support	• Methods JJ systems use to help families overcome barriers to participation	• Transportation assistance • Childcare assistance • Flexible scheduling
8. Family Health and Functioning	• Interventions delivered to families to address family behavioral health concerns • Interventions to improve family functioning and parent–child relationships	• Family therapy (Van der Pol et al.) • Parenting education and skills programs (Slavet et al., 2005)

Despite the demonstrated effectiveness of family engagement, adoption of family engagement strategies, including FDC, in JJ systems has lagged decades behind healthcare and educational settings. In fact, the vast majority of family engagement frameworks (besides FDC) were specifically developed for either pediatric or educational settings [e.g., Family-Centered Care (Johnson & Abraham, 2012), Family and Community Engagement (PFCE) Framework (U.S. U.S., 2018 and Epstein’s Six Types of Parent Involvement (Epstein et al., 2018)], with little guidance for engaging justice-involved families. Therefore, uptake has been slow and highly variable in JJ systems (Piper, Pankow, & Wood, 2023). One recent survey of JJ agencies in the U.S. suggested that JJ systems have made steps to increase their alignment with FDC domains. For instance, out of 195 JJ agencies across the US about 35% now have formalized policies to encourage family engagement in service provision (Robertson et al., 2019). The most common family engagement strategies utilized in JJ agencies included family therapy (70%), referrals to parenting skills programs (79%), and utilization of flexible scheduling to accommodate families (64%). However, many of the elements of FDC are infrequently adopted across JJ agencies including, assisting families with transportation (49%), addressing the cultural, linguistic, and sexual orientation of families (37%), inviting families to serve on advisory boards (16%), assisting families with childcare (11%), providing family support groups (7%), and providing family education groups (4%) (Robertson et al., 2019).

Due to the lack of research on strategies to improve family engagement in the legal system, studies are needed to characterize the landscape of FDC adoption in JJ agencies. To fill this gap, we evaluated current strengths and gaps in FDC adoption across 61 JJ agencies in the state of Georgia. The goals of this mixed methods evaluation were twofold: (1) assess staff-perceived strengths and gaps in current adoption of each FDC domain, and (2) assess differences in perceived adoption across organizational and staff characteristics. This evaluation was designed to provide targeted recommendations for enhancing FDC implementation within the state of Georgia and contribute to national priorities for integrating family engagement into the legal system.

## Methods

### Study design

Using an explanatory, sequential, mixed methods research design, we conducted surveys and in-depth interviews with JJ professionals in the state of Georgia to understand their perceived adoption and utilization of FDC domains in JJ agencies. Online quantitative surveys were conducted from November 2021 to February 2022, followed by qualitative key informant interviews (March- July of 2022) to supplement and clarify the quantitative findings. The study received ethical approval from both the Emory University Institutional Review Board and the Georgia JJ system research review committee.

### JJ system context

This project targets a stakeholder-identified priority area (e.g., family engagement) in collaboration with the Georgia Department of Juvenile Justice (GDJJ). Family engagement is a high priority for GDJJ and a component of their strategic plan (Georgia Department of Juvenile Justice, 2021). Families in Georgia also expressed interest in improving engagement: 100% of surveyed justice-involved families in the state wanted to be involved in developing their child’s treatment plan, and 98% expressed interest in participating in family programs (Forde & Schwartz, 2020).

Each day, approximately 7,000 youth are served at the 78 community services offices and 25 detention facilities across the state of Georgia (Georgia Department of Juvenile Justice, 2021), where GDJJ provides strengths-based, evidence-based programs to improve youth behavioral health and long-term success, including family-based programs and treatments. On an average day in GDJJ, 39% of youth are 17 years or older, 37% are 15 or 16 years old, and 14% are 14 and under. Most youth are male (70%), and 30% are female. Approximately 51% of youth are Black or African American, 39% are White, 7% are Hispanic, and 3% are another race/ethnicity. Youth can be placed in long-term secure custody, short-term incarceration, and/or community probation and diversion programs. Most justice-involved youth in Georgia have community placements (91%) and are living at home with their families (Georgia Department of Juvenile Justice, 2021).

### Participant recruitment

To recruit participants for the study, state-level JJ leaders emailed a recruitment flyer with a link to the online survey to site leaders at each of facilities in the state of Georgia (78 community services offices and 25 detention centers). Site leaders were invited to participate in the study and were asked to disseminate the survey to eligible employees at their facility. Eligible employees included JJ staff with selected roles across several divisions in GDJJ including community services (e.g., probation officers and case managers), reentry services (e.g., reentry specialists and coordinators), detention (e.g., correctional officers), education (e.g., teachers), behavioral health (e.g., providers), and administration (e.g., division directors, administrators, and managers with organizational decision/policy-making authority). These roles were selected to gain insights from staff who directly interact with families and youth, as well as perspectives from leadership who are responsible for setting family engagement agendas and promoting policy/programmatic changes. Monthly reminder emails were sent during the recruitment period, and targeted emails were sent to divisions/roles with low participation. All participants provided consent prior to completing the self-administered online survey, which took approximately 10–15 min. The survey data were collected and managed using REDCap Software (Harris et al., 2009). Overall, 140 staff and leaders from 61 agencies participated in the survey. Participants represented all key family-facing divisions (community services, reentry, detention, education, behavioral health, administration), as well as 16 of the 25 detention centers (64%) and 45 of the 78 community supervision offices in the state (58%).

As part of the study's survey, participants were asked to indicate their willingness to engage in a follow-up qualitative interview conducted over Zoom. Out of the 140 survey participants representing 61 unique agencies, 30 individuals agreed to participate in the interview. From these participants who agreed to be interviewed, we purposively selected participants representing different roles and divisions, and continued interviewing until data saturation was achieved. In total, 16 participants, consisting of 9 juvenile justice staff and 7 leaders from 10 unique agencies, participated in the follow-up interview. To incentivize participation, we donated $10 to a mental health charity for each completed interview and $5 for each completed survey.

### Measures and data collection

#### Survey

The survey measured participants’ perceived adoption of FDC strategies across the eight domains (identifying and involving family voices, informing families, collaborative decision-making and treatment planning, family diversity and inclusion, organizational feedback and policy making, family peer support, logistical support, and family health and functioning). There were 27 total survey items that measured perceived family engagement strategy adoption. Questions were adapted from and the Family System Engagement Index, which has shown good reliability in the JJ setting (Robertson et al., 2019), as well as from the principles of FDC as developed by SAMHSA and the Federation of Families for Children’s Mental Health (National Federation of Families for Childrens Mental Health, 2008). All survey items were measured on a 5-point Likert scale (1 = strongly disagree; 5 = strongly agree). The survey also collected participant demographics (including age, gender, race, ethnicity, role, years of experience, education level, and caseload) and organizational characteristics (including urbanicity, agency type (i.e., detention or community services). JJ collaborators reviewed and provided feedback on the survey and measures prior to recruitment and survey administration.

#### Interviews

The semi-structured interview guide questions assessed potential barriers and facilitators to integrating FDC domains into the JJ system. The guide focused on the eight FDC domains that were measured in the survey. Questions explored staff perspectives on current family engagement practices, recommendations regarding expanding family engagement strategies, and challenges to adopting FDC strategies in the JJ settings (see Additional File 1 for full interview guide). JJ stakeholders reviewed and provided feedback on the guide prior before starting interviews. The interview guide was iteratively adapted as interviews were conducted to improve question wording and add clarifying probes; but the core questions of the guide were not changed. Interviews were conducted online via Zoom (Zoom Video Communications Inc, 2023) by a female, qualitatively trained researcher. Interviews ranged from 25–45 min, and all participants provided verbal informed consent. All interviews were audio recorded and transcribed verbatim.

### Data analysis

#### Survey

We performed descriptive statistics (including means, standard deviations, counts, and percentages) on each item included in the survey, to assess perceived adoption of each FDC strategy. We labeled domains as “high perceived adoption” when the majority of participants agreed/strongly agreed that domain was adopted in their agency. We also performed bivariate analyses (including t-tests and one-way ANOVAs) in SPSS version 28 (IBM Corp, 2020) to assess if perceived adoption of each family engagement strategy differed across staff and organizational characteristics.

#### Qualitative and mixed methods analysis

Qualitative data were then analyzed to support interpretation of the quantitative findings. Using MAXQDA version 22.4.1 (VERBI Software, 2022), we employed standard qualitative data analysis methods including reading of transcripts, creation of a codebook, coding and consensus meetings (Hennink, Hutter, & Bailey, 2011). The codebook was developed deductively based on the eight FDC domains. Two analysts individually coded each transcript and met biweekly to discuss conflicts and ensure inter-coder agreement. Based on discussion between analysts, memos were created to summarize salient themes surrounding each domain, and we noted any strengths/weaknesses and barriers/facilitators to domain implementation. To compare the survey and interview findings, a mixed methods matrix was created to summarize findings for each domain across each data source (See Additional File 2 for mixed methods matrix). We also stratified qualitative interviews by staff and organizational demographics, to explain significant differences in adoption by agency/staff characteristics. Results were shared with JJ collaborators to ensure appropriate interpretation of findings.

## Results

Overall, 140 JJ employees from 61 different agencies in Georgia participated in the survey. On average, participants were 47 years old, most were female (*n* = 91, 71%), and most identified as Black/African American (*n* = 69, 58%) and Non-Hispanic (*n* = 117, 95%). Approximately 37% (*n* = 47) held a graduate degree and 45% (*n* = 63) worked at their agency for more than 10 years. Participants held roles including line staff (e.g., correctional officers and probation/parole officers) (*n* = 50, 35.7%), behavioral and social service providers (*n* = 26, 18.6%), educators (*n* = 26, 18.6%), case managers and reentry planning team members (*n* = 20, 14, 3%), and leadership (*n* = 18, 12.9%). Participants worked within community supervision (*n* = 73, 52.1%), detention (*n* = 48, 34.2%), and administrative (*n* = 19, 13.6%) settings, which were located in both urban (*n* = 79, 56.4%) and rural locations (*n* = 61, 43.6%).

In total, 16 JJ employees from 10 unique agencies participated in follow-up qualitative interviews, of which, 44% (*n* = 7) held a leadership role and 56% (*n* = 9) held a staff role. On average, participants were 52 years old, and most identified as Black/African American (*n* = 11, 69%) and female (*n* = 11, 69%). Participants were highly educated and experienced, with most having a graduate degree (*n* = 12, 75%) and more than 5 years of experience in JJ settings (*n* = 13, 81%). Participants represented multiple JJ divisions including reentry services (*n* = 6, 38%), community services (*n* = 5, 31%), behavioral health (*n* = 3, 19%), and education (*n* = 2, 13%). The majority of participants (*n* = 12, 75%) were located in urban areas.

Table 2 displays the distribution of survey responses (strongly disagree- strongly agree) across FDC domains and strategies. Domains with the highest perceived adoption across agencies included identifying and involving families, informing families, collaborative decision-making and treatment planning, and family diversity and inclusion. Other domains that had mixed or lower perceived adoption included involving families in organizational feedback and policy making, family peer support, logistical support, and family health and functioning. Table 3 depicts differences in perceived FDC strategy adoption by organizational- and staff-level characteristics (i.e., agency type [community supervision or detention] and staff type [leadership, behavioral health, case managers/reentry team, education, line staff]). Perceived adoption of 5 of the 8 FDC domains significantly differed across agency type (e.g., community supervision versus detention), and 3 of the 8 domains significantly differed across staff roles. In general, staff who worked in community supervision settings endorsed significantly higher adoption of the FDC domains. Strategies did not significantly differ across urban/rural, staff caseload, experience level, age, gender, or race/ethnicity (data not shown). Below we discuss the mixed methods findings for each FDC domain with joint presentation of survey data and interviewee quotes. Findings depicted in Table 2 and 3 are described within subsections below.

**Table 2.**
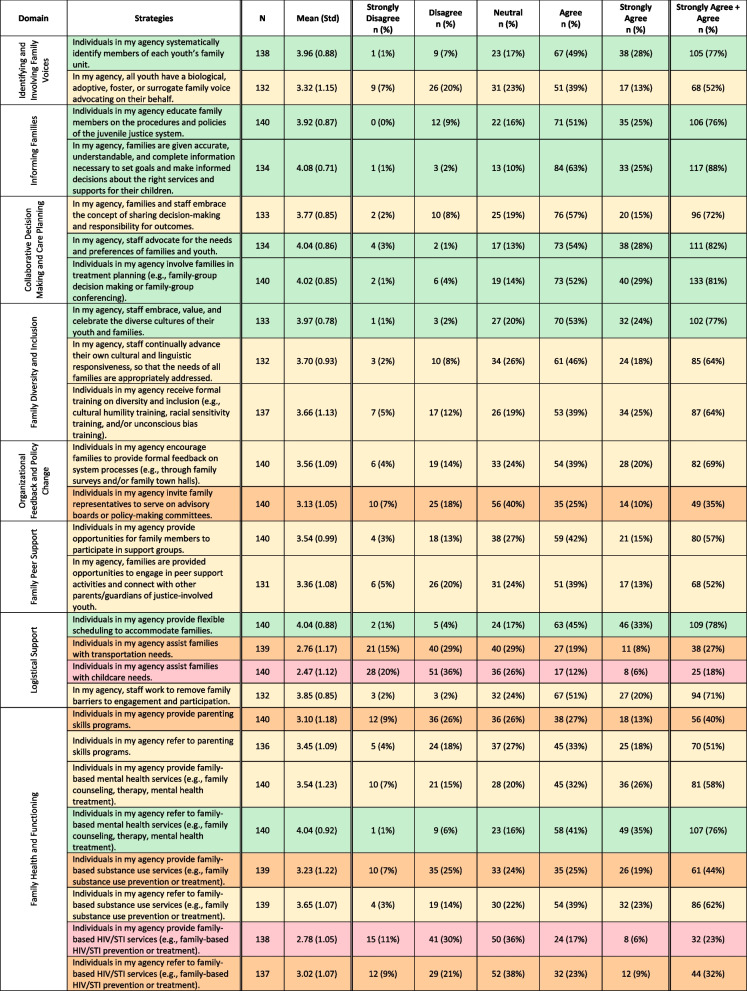
Distribution of survey responses across family-driven care domains and strategies

Row color corresponds to the percentage of participants that responded strongly agree/agree to each of the family engagement measures: green (75–100%), light yellow (50–75%), dark orange (25–50%), and red (0–25%)

**Table 3:** Relationships between participant characteristics and family-driven care implementation

Domain	Strategy	Agency Setting			Staff Role					
		Community Services *n* = 73	Detention *n* = 48	***p*** **-value**	Admin *n* = 18	Behavioral Health *n* = 26	Case Managers *n* = 20	Educators *n* = 26	Line Staff *n* = 50	***p*** **-value**
		**M(SD)**	**M(SD)**		**M(SD)**	**M(SD)**	**M(SD)**	**M(SD)**	**M(SD)**	
Identifying and Involving Families	Individuals in my agency systematically identify members of each youth’s family unit	4.04 (0.85)	3.81 (0.97)	0.17	3.83 (0.98)	4.00 (0.84)	4.16 (0.60)	3.68 (1.06)	4.04 (0.83)	0.36
	In my agency, all youth have a biological, adoptive, foster, or surrogate family voice advocating on their behalf	3.27 (1.24)	3.57 (0.91)	0.15	2.82 (1.18)	3.36 (1.03)	3.11 (1.24)	3.56 (1.00)	3.43 (1.22)	0.25
Informing Families	Individuals in my agency educate family members on the procedures and policies of the juvenile justice system	3.93 (0.95)	3.92 (0.71)	0.92	3.83 (0.70)	3.69 (0.92)	3.70 (1.12)	4.00 (0.80)	4.12 (0.77)	0.19
	In my agency, families are given accurate, understandable, and complete information necessary to set goals and make informed decisions about the right services and supports for their children	4.17 (0.64)	4.00 (0.60)	0.15	3.94 (0.94)	4.04 (0.73)	4.15 (0.74)	4.08 (0.57)	4.13 (0.65)	0.88
Collaborative Decision Making and Care Planning	In my agency, families and staff embrace the concept of sharing decision-making and responsibility for outcomes	3.88 (0.88)	3.62 (0.77)	0.1	3.44 (0.92)	3.68 (0.85)	4.05 (0.88)	3.71 (0.75)	3.85 (0.84)	0.23
	In my agency, staff advocate for the needs and preferences of families and youth	4.13 (0.82)	3.91 (0.89)	0.18	3.56 (0.92)	4.24 (0.66)	4.30 (0.57)	3.96 (0.97)	4.04 (0.91)	0.06
	Individuals in my agency involve families in treatment planning (e.g., family-group decision making or family-group conferencing)	4.27 (0.65)	3.65 (0.91)	** < 0.001**	3.89 (1.07)	3.81 (0.69)	4.25 (0.55)	3.54 (1.02)	4.34 (0.68)	** < 0.001**
Family Diversity and Inclusion	In my agency, staff embrace, value, and celebrate the diverse cultures of their youth and families	4.10 (0.74)	3.80 (0.77)	**0.04**	3.89 (0.67)	3.71 (0.90)	4.05 (0.88)	4.00 (0.70)	4.09 (0.73)	0.38
	In my agency, staff continually advance their own cultural and linguistic responsiveness, so that the needs of all families are appropriately addressed	3.82 (0.94)	3.60 (0.84)	0.2	3.44 (0.98)	3.60 (1.0)	3.90 (0.72)	3.68 (0.85)	3.80 (1.00)	0.55
	Individuals in my agency receive formal training on diversity and inclusion (e.g., cultural humility training, racial sensitivity training, and/or unconscious bias training)	3.86 (1.08)	3.41 (1.17)	**0.03**	3.59 (1.18)	3.58 (1.23)	3.95 (1.23)	3.40 (0.91)	3.73 (1.13)	0.56
Organizational Feedback and Policy Change	Individuals in my agency encourage families to provide formal feedback on system processes (e.g., through family surveys and/or family town halls)	3.53 (1.1)	3.71 (1.01)	0.38	3.44 (1.24)	3.50 (0.94)	3.50 (1.1)	3.88 (0.95)	3.50 (1.16)	0.59
	Individuals in my agency invite family representatives to serve on advisory boards or policy-making committees	2.92 (1.01)	3.40 (1.09)	**0.007**	3.17 (1.15)	3.08 (0.84)	2.95 (0.88)	3.58 (1.10)	2.98 (1.11)	0.17
Family Peer Support	Individuals in my agency provide opportunities for family members to participate in support groups	3.59 (1.04)	3.40 (0.92)	0.29	3.67 (0.97)	3.35 (0.97)	3.45 (0.99)	3.46 (0.91)	3.66 (1.06)	0.67
	In my agency, families are provided opportunities to engage in peer support activities and connect with other parents/guardians of justice-involved youth	3.33 (1.16)	3.38 (0.98)	0.82	3.06 (1.06)	3.36 (1.03)	3.45 (1.23)	3.42 (1.01)	3.39 (1.10)	0.84
Logistical Support	Individuals in my agency provide flexible scheduling to accommodate families	4.36 (0.61)	3.48 (1.01)	** < 0.001**	4.22 (0.73)	3.81 (1.16)	4.20 (0.61)	3.50 (0.99)	4.32 (0.62)	** < 0.001**
	Individuals in my agency assist families with transportation needs	2.88 (1.25)	2.65 (1.06)	0.3	3.00 (1.23)	2.52 (1.16)	2.45 (1.19)	2.81 (1.05)	2.90 (1.82)	0.41
	Individuals in my agency assist families with childcare needs	2.56 (1.26)	2.48 (0.92)	0.69	2.33 (1.13)	2.15 (0.92)	2.20 (1.00)	2.65 (0.84)	2.70 (1.31)	0.17
	In my agency, staff work to remove family barriers to engagement and participation	3.94 (0.79)	3.73 (0.81)	0.18	3.83 (0.92)	3.80 (0.76)	3.79 (0.78)	3.79 (0.93)	3.93 (0.87)	0.94
Family Health and Functioning	Individuals in my agency provide parenting skills programs	3.12 (1.24)	3.02 (1.12)	0.64	3.22 (1.35)	2.81 (1.02)	2.90 (1.11)	3.19 (1.05)	3.24 (1.27)	0.52
	Individuals in my agency refer to parenting skills programs	3.59 (1.16)	3.16 (0.94)	**0.04**	3.56 (1.19)	3.25 (0.98)	3.65 (1.18)	3.13 (0.94)	3.58 (1.12)	0.35
	Individuals in my agency provide family-based mental health services (e.g., family counseling, therapy, mental health treatment)	3.71 (1.29)	3.35 (1.10)	0.35	3.33 (1.13)	3.50 (1.10)	3.65 (1.26)	3.27 (1.15)	3.74 (1.35)	0.51
	Individuals in my agency refer to family-based mental health services (e.g., family counseling, therapy, mental health treatment)	4.30 (0.78)	3.52 (0.98)	** < 0.001**	4.06 (0.87)	3.88 (0.86)	4.35 (0.81)	3.42 (1.03)	4.30 (0.79)	** < 0.001**
	Individuals in my agency provide family-based substance use services (e.g., family substance use prevention or treatment)	3.29 (1.34)	3.25 (1.08)	0.89	3.00 (1.13)	3.35 (1.09)	3.15 (1.30)	3.04 (0.95)	3.39 (1.41)	0.66
	Individuals in my agency refer to family-based substance use services (e.g., family substance use prevention or treatment)	3.88 (1.13)	3.35 (0.91)	**0.009**	3.44 (1.04)	3.50 (0.94)	3.85 (1.08)	3.23 (0.91)	3.96 (1.13)	**0.03**
	Individuals in my agency provide family-based HIV/STI services (e.g., family-based HIV/STI prevention or treatment)	2.66 (1.12)	3.00 (1.03)	0.09	2.89 (0.96)	2.73 (0.96)	2.45 (0.99)	3.04 (0.91)	2.75 (1.19)	0.42
	Individuals in my agency refer to family-based HIV/STI services (e.g., family-based HIV/STI prevention or treatment)	2.99 (1.19)	2.98 (1.0)	0.97	3.22 (0.88)	2.88 (0.95)	3.00 (1.2)	3.00 (1.0)	3.04 (1.21)	0.9

Shading indicates a significant relationship at the *p* < 0.05 level

### Identifying and involving family voices

The majority of survey participants (77%) agreed that “JJ staff work with youth to identify members of their family unit” (Table 2). Similarly, interview participants explained that parents and family members were identified from the very beginning of youth's encounter with the justice system; and when parents/guardians are unable to participate, JJ staff work with the child to identify and involve other members of the family unit (such as siblings, grandparents, and other extended family members) who can serve as advocates and support systems throughout their time in the JJ system:

*"When you say family engagement, it means involving anybody that touches the youth, that they feel important in their life. And so that’s not necessarily confined to just a biological parent – But it’s guardians or somebody that – or extended family members as well as important people to the family, that they regard as family."* (ID: #15, Leadership, Reentry Services)

Although staff attempt to involve family members in all processes and decisions, only 52% of participants agreed/strongly agreed that all youth have a family voice advocating on their behalf (Table 2). Interviewees noted that some families are unable to engage in JJ processes (due to competing demands, financial insecurity, or other logistical barriers like transportation), or they are unwilling to engage with JJ staff (due to mistrust and strained interpersonal relationships with staff). As expressed by interviewed participants, many believed that having family voices involved in all steps of the service provision process is directly linked to youth’s success:

*“If we have the parent buy in, their support, I’ve seen it where the parent from the time we do their intake appointment, all the way through the whole process that we’re supervising that case, their buy in and listening and being able to have that open line of communication, to make sure that the kid gets everything they need to get them back on track is key and when we don’t have that parent buy in, it can be a barrier sometimes….If we don’t have the buy in from the parent, that’s going to be a barrier to getting kids to groups or getting the kids to counseling appointments, getting the kid whatever resources they need."* (ID: #1, Leadership, Community Services)

Overall, perceived adoption of this domain did not differ between JJ settings (e.g., community supervision versus detention) and staff roles (e.g., admin, behavioral health, education, case managers, line staff) (Table 3).

### Informing families

The majority (88%) of survey participants agreed/strongly agreed that “families are given accurate, understandable, and complete information”; and 76% of participants agreed/strongly agreed that “JJ staff educate family members on the procedures and policies of the JJ system” (Table 2). There were no significant differences in perceived adoption by staff or agency characteristics (Table 3). In alignment with survey findings, interviewees discussed the strong adoption of this domain:

*“[We develop] a relationship where there is communication between families and accessibility, where families have information and can email as well as call staff who work directly with their kid. They’re informed about if something happens within a certain timeframe and policies are in place to support that, so it’s not like an individual’s decision about how to make that happen. There are policies that are out there…I think we’re doing really good with communication and transparency and keeping them involved and informed.”* (ID: #16, Staff, Reentry Services).

Participants discussed how families are informed at all steps in the process, and there are policies in place to ensure they are informed in a timely, transparent, and accurate manner, starting with an initial intake and orientation meeting:

*“One of the measures that we use is we have a required standards of contact [with families] – depending on the child’s arrest level or their level of supervision and based on that standards of contact, we have an intake appointment with the families and explain to them what the expectations are, to ensure that we’re all on the same page in regards to how often the child needs to be seen, how often do we need to see the parent, how many phone calls we need to make, collaborations and collaborative meetings. So that’s one of the measures that we use agency wide in regards to how we communicate with the families, just having that initial intake appointment for them to understand that.”* (ID: #1, Leadership, Community Services)

After the intake and orientation meeting, families are continuously informed of their child’s progress and expectations for the remainder of their involvement with the system:

*“They have meetings where the parents are involved in those…they’re done like every 30 days. So the parents are usually on the call along with the probation officer, the counselors. So they talk about expectations leading up to the child getting out and the things that are expected once that youth is released.”* (ID: #8, Staff, Behavioral Health)

Staff expressed that keeping families up to date with any new information is a priority and supported by policies within the department. In addition to face-to-face meetings, staff utilize various modes of communication including email, text, call, or videoconferencing to keep the family informed. They also provided informational brochures on JJ system procedures as well as informational materials related to resources and services available in their community.

### Collaborative decision making and care planning

Overall, 81% of survey participants agreed/strongly agreed that “staff involve families in treatment planning”, 82% agreed/strongly agreed that “staff advocate for the preferences of families”, and 72% agreed/strongly agreed that “families and staff embrace the concept of shared decision-making” (Table 2). All interview participants discussed how collaborative care decisions, including decisions related to behavioral health treatment and prevention services, were standard practice in their system:

*“It’s become standard… the new staff see it as part and parcel of how we do business. All our staff are trained in motivational interviewing. The central theme in the use of motivational interviewing is to ensure that family and youth opinions are solicited, decisions are made and supported whenever can, and that they feel as equal partners in the process. And I would not hesitate to say that, without hard numbers to back me up, that [families] do find that they are partners and not just subjects of what we do.”* (ID: #11, Staff, Reentry Services)

Although this domain is strong across all settings and staff types, collaborative care planning received significantly higher endorsement from surveyed staff in community supervision settings compared to detention settings (mean = 4.27 in community supervision versus 3.65 in detention), as well as higher endorsement from case managers/reentry team members (mean = 4.25) and line staff (mean = 4.34) compared to other staff types (mean = 3.89 [admin], 3.81 [behavioral health providers], 3.54 [educators]) (Table 3). Interview participants discussed how collaborative care planning is a high priority, especially for youth transitioning back home to their families, which may explain higher endorsement among community supervision and reentry staff:

*“Because if the family’s not involved, how can you welcome their child back home? You’ve got to have [the family] involved in the consultation and the discussion of the care and the discussion of the education and discussion of the medication. They’ve got to be involved. If you continue to involve them, then they’re more receptive to their child coming back home- somebody else has been raising them for that timeframe- and to keep them a part of the conversation, then that has helped out with them being ready to accept the kid coming back home.”* (ID: #16, Staff, Reentry Services)

### Family diversity and inclusion

The majority of survey participants (77%) agreed that “staff embrace, value, and celebrate the diverse cultures of their youth and families” (Table 2). This statement was more likely to be endorsed by staff from community supervision settings (mean = 4.10 in community supervision versus 3.80 in detention settings) (Table 3).In addition, 64% agreed/strongly agreed that “staff continually advance their own cultural and linguistic responsiveness”, and 64% agreed/strongly agreed that “staff receive formal training on diversity and inclusion” (Table 2). Similar to survey findings, interview participants discussed how staff (especially those in community supervision settings) are continually trained on diversity, equity, and inclusion principles: *“We definitely train our staff in terms of cultural sensitivity, cultural implicit bias training, things of that nature, as we work with our youth and our families.”* (ID: #2, Leadership, Reentry Services). Additionally, participants discussed how the department focuses on hiring staff from diverse backgrounds to promote representation:

“*By making sure our team is racially diverse, number one. And making sure that we understand that we have to be sensitive and aware of racial diversity. But I think when you’ve got people that come from diverse communities, they have a more personal understanding about what that really means*.” (ID: #15, Leadership, Reentry Services)

In addition to training and promoting workforce diversity, the department also has mechanisms to monitor inequities in the system and respond to family grievances:

*“We also are trying to look at making sure that we’re not creating any disparities in our health services or any of the other services that we do because we have a quality assurance monitoring program where we look at health services. … And there’s a grievance system for young people to utilize. So there are several mechanisms that both the youth can utilize and the parent can utilize when they feel like they’re not being treated fairly.”* (ID: #6, Leadership, Behavioral Health)

### Organizational feedback and policy change

The majority of survey participants (69%) agreed/strongly agreed that “families are encouraged to provide feedback on system processes” (Table 2). Interviewees discussed how families are invited to provide feedback on organizational procedures through several methods, including through the Ombudsman office, through family surveys, and during family roundtable sessions (called The Chat): *“we have a family engagement call that allows families to come and hear about our resources, as well as room for them to bring up any concerns that they may have”* (ID: #10, Staff, Reentry Services). Staff also hoped that families feel comfortable approaching them with any concerns, and many line staff check-in with families regularly through phone calls or collaborative care meetings:

*“They certainly have opportunity to give feedback to me. It’s not a thing of them having to figure out how do I do this. I go after it. I will ask them, what’s working, what’s not, what do we need to change, how can we adapt?”* (ID: #4, Staff, Community Services)

However, only 35% of survey participants agreed/strongly agreed that “families are invited to serve on advisory boards or policy making committees.” Strategies to involve families in organizational policy-making were more commonly endorsed by staff in detention settings compared to community supervision settings (mean = 3.40 in detention versus 2.92 in community supervision) (Table 3). Unlike community supervision settings, participants discussed how detention facilities had community advisory groups: “*at every facility, there’s like an advisory board from the community that we involve parents and folks In the community for that”* (ID: #6, Leadership, Behavioral Health). In addition, participants noted that JJ schools within the facilities used family advisory boards to inform their educational activities “*So we have a whole separate arm of family engagement going on through the school system, focusing on the school system, focusing on education. They have actually stood up a family advisory committee within their unit*” (ID: #11, Staff, Reentry Services).

Despite these local efforts (mainly within the detention setting), staff hoped that a system-wide family advisory board would be established soon: *“We’ve been working on a parent advisory committee. I would love to see us have one of those where we have parents involved around the table to tell us how we can better serve them.”* (ID: #2, Leadership, Reentry Services) However, concerns about meeting accessibility and family compensation are creating obstacles to advisory board implementation:

*“We also have to realize that some of our families have had traumatic experiences with government agencies and so we understand that it’s important to build trust with families, before we can really engage them in certain ways, and to get families to open up to us and to inform policy and procedures, we first have to build a relationship with them and build trust in order for them to feel comfortable doing that.….And so if I’m asking you to come participate in a family advisory committee, you may have three little ones at home, so I may need to help provide child care so that you can be available. I may need to give you a gift card or something to have pizza brought in. We’re not paying families to partner with us, but we understand that families have specific needs and if we really want to engage with them and glean valuable information from them, then there needs to be something on our side that says we know that this may present a hardship and here’s something to help with that.”* (ID: #12, Leadership, Reentry Services)

### Family peer support

Overall, 52% of survey participants agreed/strongly agreed that “families are provided opportunities to engage in peer support activities,” and 57% agreed/strongly agreed that “families are provided opportunities to participate in support groups” (Table 2). Interviewees described a few events which provide an avenue for families to connect with one another, including family meetings where JJ professionals provide resources, classes, and educational materials for parents and guardians:

*“From time to time do have different support groups, parents or family members identify things that are concerning, we make sure we follow up and try to address those concerns. It’s not just limited to parents who have youth still in secure confinement, but anybody that wants to come into that chat or anybody that might have – have a youth that’s been involved at some point and through those chats, we also provide classes and different presentations for the participants. And each month we might focus on something different. We’ve had cooking classes where they would cook and got certain foods and utensils at the end of the course.” (ID: #15, Leadership, Reentry Services)*

Interview participants expressed interest in adopting more family peer support programs, including a parent peer counseling group that was previously offered:

*“We had a mental health counselor who came in on weekends during visitation and those parents who wished to participate could stay for a parent counseling group. And, yeah, the parents loved it, you know, and it was kind of part counseling, part planning and part peer support. And the parents just loved it. I would love to be able to offer something like that at all of the 25 facilities, you know, and have staff who are dedicated to coming in on the weekends when it’s convenient for the parents and doing that.”* (ID: #7, Leadership, Behavioral Health)

### Logistical support

The majority (78%) of participants agreed/strongly agreed that “staff provide flexible scheduling to accommodate families” (Table 2). The perceived adoption of flexible scheduling significantly differed across organizational and staff characteristics. For instance, flexible scheduling was more likely to be endorsed by staff working in community supervision settings compared to detention settings (mean = 4.30 in community supervision and 3.52 in detention), and more likely to be endorsed by line staff (mean = 4.32), case managers/reentry team members (mean = 4.20), and admin (mean = 4.22) compared to other staff types (mean = 3.81 [behavioral health] and 3.50 [education]) (Table 3). In addition to scheduling, 71% of participants agreed/strongly agreed that “staff work to remove family barriers to engagement and participation” (Table 2). This schedule flexibility and increased accessibility was especially evident during the COVID-19 pandemic, where staff removed participation barriers by utilizing virtual communication channels.

*“We were very creative in continuing the work that we do with our families during especially, you know, at the height of the COVID situation. A lot of providers even went to virtual therapy. Our parents, we purchased iPads for the youth so that we could do visitation virtually in a lot of cases. There was virtual courts that we coordinated at the facility with the court system. We learned how to utilize technology quite a bit more. And so we learned that that’s one way to allow one of our youths who parents may not be able to necessarily visit with them as much but virtually – they could communicate and have some time with their youth.” (ID: #2, Leadership, Reentry Services)*

In addition to utilizing virtual communication, participants also discussed other ways community supervision staff helped families overcome logistical challenges, such as through helping them schedule and access referral appointments:

*“But another major challenge I think is helping not just the youth, but recognizing that there’s a family situation that you need to assist them with and like I said, transportation is one of those things. Making sure that they have the things that are needed as it relates to being able to engage in those services, like proper identification. We have programs for that. Making sure that they’re not just told where their appointments or referrals are, but helping them make those appointments and facilitating the process to make sure they’re able to get there.” (ID: #15, Leadership, Reentry Services)*

Despite efforts to overcome logistical barriers to family participation, only 27% of survey participants agreed/strongly agreed that “staff assist families with transportation needs” and only 18% agreed/strongly agreed that “staff help families with childcare needs” (Table 2). Participants recognized these that there are gaps in their ability to help families overcome participation barriers such as childcare and transportation:

*“We make stuff available, but it’s very difficult for families to engage in those processes. They have to work, just trying to find the right time when we make stuff available... If it’s in the evening, they worked all day and now they’re trying to take care of their families. And also making sure that when there are opportunities that they can be in person for certain things, making sure we have some resources available to help deal with any type of challenges they may have as it relates to transportation. We can sometimes help with those things. And the biggest thing is when a person is released and got appointments, especially with doctors and mental health appointments, if they can’t get to them, that’s a real barrier. And a lot of times – we might can get the youth there, but the transportation does not allow for a family member to access that transportation. And if the parent can’t go with them, guess what, they don’t end up going. The transportation, those are the biggest challenges we have in terms of how do we find and provide services.”* (ID: #11, Staff, Reentry Services)

### Family health and functioning

Less than half of participants (40%) agreed/strongly agreed that staff “provide parenting skills programs”, and 51% agreed/strongly agreed that staff “refer to parenting skills programs” (Table 2). Due to their role in case management and service linkage, staff in community supervision settings (mean = 3.59 for community supervision versus 3.16 for detention) were more likely to endorse referrals to parenting programs. Staff indicated that they offered some parenting skills programs onsite, but they needed to increase family participation: *“So there are classes available for [family]. But yet again, it’s a matter of [family] participation”* (118). Their parenting skills program (called Family Café) utilizes an evidence-base curriculum, which involves a series of presentations and workshops for families to learn skills that can be applied when their children return home:

*“We do offer some programs and supports through initiatives like the Family Café that we offer, which uses an evidence-based curriculum and teaches Active Parenting. We offer a nurturing parenting curriculum for our youth and families…But it’s just interesting to really have a conversation with parents and talk about some of the challenges and barriers, and for parents to know that people like me and others on our team, who are also parents, we’re all going through the same things with our kids and it’s the brainstorming and networking and building relationships and people let their guard down, we’re not seen as law enforcement, we’re seen as a fellow parent.” (ID: #12, Leadership, Reentry Services)*

In addition to parenting skills programs, staff also offered family-based mental health services, including family counseling and therapy. Most survey participants (76%) agreed/strongly agreed that “family-based mental health services were offered via referral to external community-based providers”, while 58% agreed/strongly agreed that “family-based mental health services were offered on-site”. Additionally, 62% of participants agreed/strongly agreed that” families are referred to family-based substance use services” and 44% agreed/strongly agreed that “family-based substance use services are provided on-site” (Table 2). Referrals to family-based mental health and substance use providers were more commonly endorsed by staff from community supervision agencies and line staff/case managers (Table 3). A participant described the different family-focused mental health and substance use services offered by reentry community services staff:

*“Typically we use services that are kind of wrap around services. So we’ll have like one vendor that will be able to address the individual counseling, the family therapy, as well as like substance abuse and other things that the individual may need, as well as stuff like MST, which is the multisystemic therapy. And they also work with the family as well as the individual youth to teach them coping mechanisms as well as life skills and things like that. We also utilize mentoring, in which they kind of engage in the family as well, to kind of make sure that the individuals are moving towards their more prosocial activity aspect as well as like I said, those life skills that are really important.”* (ID: #5, Staff, Community Services)

Although referrals to family-based mental health and substance use providers are commonly endorsed by participants, provision of sexual health education programs, prevention services, or STI/HIV services were rarely endorsed by participants. Only 32% of participants agreed/strongly agreed that their agency “refers to family-based sexual health programs”, and only 23% “provided family-based sexual health programs on-site” (Table 2). In the interviews, participants did not discuss any sexual health programs that involved both youth and families.

## Discussion

In this evaluation of FDC implementation in JJ agencies across Georgia, we identified key strengths and gaps in current adoption. Domains with the highest perceived adoption across agencies included identifying and involving families in all processes, informing families, collaborative decision-making and treatment planning, and family diversity and inclusion. Other domains that had mixed or lower perceived adoption included organizational feedback and policy making, family peer support, logistical support, and family health and functioning.

Adoption of FDC domains was highly variable across JJ agencies within the state; which is consistent with a prior national study that captured the heterogeneity in family engagement strategies between jurisdictions (Robertson et al., 2019). The adoption of new interventions in JJ settings is dependent on the context of local jurisdictions, including local JJ policies, structure, culture, and resources (Becan et al., 2020; Prendergast et al., 2017; Sales et al., 2018; Taxman, Henderson, & Belenko, 2009). FDC strategies varied across staff and organizational characteristics, including staff roles and agency type. In general, FDC strategies were more likely to be adopted in community supervision/probation settings (rather than detention settings) and were most likely to be endorsed by case managers/reentry team members, which is likely because these settings and staff types meet with families regularly and have the opportunity to develop the rapport needed to implement FDC strategies. These findings suggest that community supervision agencies may have higher readiness to implement FDC, since family engagement naturally fits within the culture and workflows of those agencies. However, more research is needed to understand how to increase FDC adoption in detention settings, specifically identifying the training, support, and resources needed to improve adoption in these facilities. In addition to differences by agency type and staff roles, a prior study also identified that family engagement was higher in rural locations; however, we did not observe differences in adoption between rural and urban locations in this state (Robertson et al., 2019). Future studies should consider how geographic location (such as rural/urban characteristics) and community-level factors (such as service accessibility and availability) influence adoption of FDC practices and family participation. To ensure FDC implementation is consistent across agencies and available to all families, JJ systems should build awareness of family engagement and provide training to staff from all divisions and levels of the system.

### Current FDC adoption successes

Participants perceived high adoption of the first FDC domain, *identifying and involving family voices*. Staff described how JJ staff work with youth to identify the members of their family and community support system, which ensures that an advocate is involved in all aspects of youth’s arrest, detention, disposition, and treatment (Walker et al., 2015). Similar to Georgia, some JJ systems are using specific tools such as the Juvenile Relational Inquiry Tool (JRIT) to systematically identify strengths and gaps in the child’s support system (Shanahan & Agudelo, 2011; Shanahan & diZerega, 2016). One study reports that the JRIT increased youth’s connectedness and commitment to family members (Shanahan & Agudelo, 2012). Other methods to identify family networks include genograms and ecomaps, which are visual tools that map the interpersonal relationships present in the youth’s life, as well as depict roles, patterns of communication, and social interactions between family members. In child mental health settings, the use of genograms significantly increased family engagement and retention in behavioral health treatment (Coatsworth et al., 2001; Dakof et al., 2003; Santisteban et al., 1996; Szapocznik et al., 1988), but more research is needed to understand its utilization and effectiveness in JJ settings.

Another domain that was strongly adopted across agencies was *informing families*. JJ staff believed they effectively communicated and shared information with families about JJ system procedures, resources, and available services and supports. This domain is critical because many family members lack the experience and knowledge necessary to navigate the JJ system (Walker et al., 2015). A national research report by justice-involved families found that the vast majority of families viewed the JJ system as very confusing, and only about 18% of families said that justice staff were helpful in assisting them with understanding the process (Justice for Families, 2012a). Similar to agencies in this study, JJ systems across the country are implementing strategies to effectively inform families, such as family orientations (Luckenbill, 2012; Osher et al., 2012). One family orientation program in Washington was shown to significantly increase justice system-related knowledge and was perceived as ‘very helpful’ by most family members (Walker, Pullmann, Trupin, Hansen, & Ague, 2011). Some states also provide tours of the detention facility during orientation to ease family members’ feelings of anxiety and to build trust between staff and families (Arya, 2013). Along with orientations, JJ organizations are distributing “family-friendly” handbooks, which provide resources, contact information, rights and responsibilities, ways to participate, and basic descriptions of the JJ system and processes (Connecticut Center for Effective Practice n.d.; Family Involvement Committee of the PA Council of Chief Juvenile Probation Officers, 2012; Smelstor, 2000). Families report that handbooks and educational materials are most helpful when they are developed in collaboration with families, and JJ advocacy organizations emphasize that materials should be translated to languages that fit the populations served (Arya, 2013; National Center for Mental Health and Juvenile Justice, 2016).

C*ollaborative decision-making and treatment planning* became standard practice among agencies in this study after recent state-level reforms and creation of the reentry services division. This domain involves partnering with families to develop treatment plans and goals for the child that reflect the needs and preferences of the family (Walker et al., 2015). Strategies such as Family Conferencing or Family Group Decision Making (FGDM) are promising methods to involve family members, youth, JJ staff, and providers in the collaborative development of a treatment action plan (Annie & Casey Foundation, 2014; Lewis & Judge, 2005; Pennsylvania’s Family Group Decision Making (FGDM) Leadership Team, 2009). Collaborative care planning strategies, such as FGDM, are based on the belief that families can provide background and context, including information on traumas and behavioral issues, which helps providers decide on treatment options. Research also suggests that families are more likely to support and implement services they helped develop (Richard Spoth & Redmond, 1993, 1995; R. Spoth et al., 2000). FDGM has been gaining popularity in JJ settings, and a prior study found that 76% of community supervision agencies reported family involvement in treatment planning and 42% reported that families are involved in choosing the level and type of treatment (Robertson et al., 2019). One pilot study conducted in JJ agencies found that FDGM increased family satisfaction with the JJ process and improved job satisfaction for staff because they developed stronger relationships with families (Pennsylvania’s Family Group Decision Making (FGDM) Leadership Team, 2009). Besides this pilot study, the majority of FDGM research was conducted in child welfare settings. These studies suggest that FDGM improves collaboration between families and professionals (Ferguson, 2004), increases family empowerment and satisfaction (Sheets et al., 2009), improves family-child connections (Pennell & Burford, 2000), increases the likelihood that youth initiate treatment services (Weigensberg et al., 2009), and decreases contacts with child protective services (Crampton, 2003; Pennell & Burford, 2000).

*Family Diversity and Inclusion* is another domain that was strongly implemented across agencies in our study. Studies have shown that youth of color (e.g., Black/African American and Hispanic/Latinx) and sexual/gender minority youth are overrepresented in the JJ system (Hanes, 2012; Loyd et al., 2019; Marrett, 2017; Poteat et al., 2016; Spinney et al., 2018). Based on data from 2018, minority youth in Georgia are significantly overrepresented in the JJ system, especially among youth that are deeper in the system (i.e., placed in secure confinements and referred to adult courts). Although African American youth represent 34% of the youth population in Georgia, they make-up 60% of referrals to the JJ system, 71% of secure confinements, and 79% of referrals to adult courts (Gonzales et al., 2018). In response to these disparities, JJ systems are taking steps to develop cultural competencies. One survey indicated that about 37% of JJ agencies actively addressed the cultural, linguistic, and sexual orientation of families (Robertson et al., 2019). Cultural and diversity trainings are growing in popularity among JJ organizations, and trainings have been shown to improve JJ staff’s cultural awareness, increase family involvement in the JJ process, and enhance families’ ability to advocate for their child (Hoytt, Schiraldi, Smith, & Ziedenberg, 2002; Willison, 2010). In addition to staff training, JJ services should be culturally adapted to fit the populations served. For instance, one JJ site in Washington adapted their Functional Family Therapy (FFT) program for African American families: the adapted program increased FFT completion rates from 45 to 100% (DMC Action Network, 2009). Other studies found that implementation of culturally adapted programs in JJ settings increased family satisfaction and improved youth treatment outcomes (Burrow-Sanchez & Wrona, 2012; Burrow-Sánchez et al., 2015; DiClemente et al., 2014). In addition to program adaptations, some JJ systems are trying to mitigate language barriers. JJ facilities in California hired more Spanish-speaking staff and partnered with Spanish-speaking family liaisons: as a result of these changes, more than twice as many youth were diverted from detention to community-based programs (Hoytt et al., 2002).

### Opportunities and future directions for improving FDC implementation

*Organizational feedback and policy making* is an area of future development for Georgia’s JJ system, to ensure family perspectives are incorporated into programmatic and policies initiatives at the state-level. Some JJ organizations across the nation are utilizing family advisory boards, where policies and practices are informed by insights from family members who are currently or formerly involved with the agency (Arya, 2013). Research suggests that advisory boards improve process outcomes such as population engagement in programs and alignment of programs with community needs (Oldfield et al., 2019). These methods to engage families in organizational decision making are becoming more common in the JJ system, and a national survey estimated that about 16% of agencies invited family representatives to serve on advisory boards (Robertson et al., 2019).

Another domain with lower levels of implementation was *family peer support*, which refers to the emotional and tangible support provided by other family members who have children in the JJ system. Peer support is an evidence-based practice: in medical settings, family peer support has been shown to increase family self-efficacy to care for their child, enhance family-professional collaboration, improve family empowerment and confidence to advocate for their child, increase family self-care, reduce internalized blame, and decrease family isolation (Hoagwood et al., 2010; Koroloff et al., 1996; Kutash et al., 2011; Leggatt & Woodhead, 2016; Obrochta et al., 2011; Purdy, 2010; Robbins et al., 2008). Specifically in the JJ setting, peer advocates in Colorado developed individualized plans for families, provided peer emotional support, attended appointments with families, and provided family support groups: this peer advocate program effectively increased youth engagement in treatment and decreased youth reoffending (Cataldo & Ford, 2010). Also, one county in Washington trained “family partners” to provide resources and emotional support to families who were entering the JJ system: the program significantly increased families’ self-efficacy to navigate the JJ process (Walker et al., 2011). Despite growing evidence, very few JJ agencies provide family peer support: a national survey found that only 7% of agencies offer family support groups (Robertson et al., 2019), suggesting this is an area of future research and development not only for Georgia but fore JJ systems nationally.

Family *logistical support* is another FDC domain with perceived weaknesses. Although staff worked with families to overcome barriers, participants believed the lack of transportation was an obstacle to family participation in Georgia. In one survey of justice-involved families, the most common barriers to participation were logistical obstacles including transportation (42%), distance (41%), time (37%), and cost (35%) (Justice for Families, 2012a). Some JJ facilities are responding to these challenges by providing flexible scheduling, transportation assistance, and childcare (Robertson et al., 2019). Although programs exist, there is limited documentation of the impact of logistical support on family or youth outcomes. In the healthcare literature, these family engagement barriers (e.g., transportation, scheduling, cost) are also commonly reported (Syed et al., 2013). In one study, adolescent mental health providers were trained to support families’ ability to initiate therapy by addressing financial, transportation, and scheduling concerns: this led to higher therapy initiation and engagement compared to the control group (McKay et al., 1996). Another study found that that provision of a car, van, or contracted transportation services improved behavioral health treatment retention (Friedmann et al., 2001). Despite a few studies, there is a paucity of research and interventions to address logistical barriers to family engagement in the JJ setting.

Lastly, *family health and functioning* include programs that address the family environment, such as family-based treatment programs (i.e., Multisystemic Therapy, Multidimensional Family Therapy, Family Behavior Therapy, and Functional Family Therapy) and parenting skills/education programs. Evidence-based interventions that provide family skills and therapy are effective in improving youth outcomes such as recidivism, mental health, and substance use (Slavet et al., 2005; Trupin et al., 2011; Van der Pol et al.; Woolfenden, Williams, & Peat, 2002). In this study, referrals to family-based mental health and substance use programs were common, especially in the community services setting. This aligns with a prior survey that found 70% of community services agencies refer to family therapy (Robertson et al., 2019). However, parenting skills programs were less commonly endorsed by participants and were identified as an area of future growth for the department. Also, sexual health programs, especially programs that involve families, are rarely adopted in Georgia and JJ settings nationally, presenting another opportunity for future growth (Tolou-Shams et al., 2010).

### Strengths and limitations

Although this study provided important data to inform the scale-up of family engagement programs in JJ agencies within Georgia and potentially nationally, it has limitations which should be considered in the interpretation of findings. First, the data were collected using non-probability sampling. Therefore, participants who volunteered for the surveys and interviews may be more attuned to family engagement processes compared to the general population of JJ staff. Although we received survey participation from all JJ divisions, 16 of the 25 detention centers, and 45 of the 78 community supervision offices, we do not have an accurate measure of the staff response rate, which limits our understanding of survey generalizability at the individual level. Additionally, there is variability in the contexts that shape delivery of services to families across jurisdictions, and our findings may not be applicable to other states; however, we hope this evaluation can serve as a roadmap for other states that are expanding their family engagement initiatives. Also, assessment of domain adoption is based on staff self-reports and not based on direct observation, so responses may not reflect actual implementation across agencies. In addition, perceived adoption skewed towards agree/strongly agree (suggesting possible social-desirability bias), and future studies are needed to confirm self-reported adoption (e.g., direct observations of system operations and practices). However, a strength of this mixed methods study is the strong corroboration between quantitative and qualitative findings, enhancing reliability of these results in the context of Georgia’s juvenile justice system. Future research is needed to design, implement, and evaluate strategies to strengthen family engagement and FDC strategies in JJ systems across the U.S. Most notably though, the majority of the limited research on this topic has focused on JJ staff, thus, future research should focus on family and youth perspectives, to develop acceptable interventions that address the needs and experiences of justice-involved families.

## Conclusions

In conclusion, engaging families in behavioral health services and JJ system processes is a high priority for systems across the country. Historically, the JJ system context has not been inclusive of family voices, but recently family advocacy and reform efforts have highlighted the critical need for family collaboration to improve youth health, behavior, and recidivism outcomes. This study highlighted the many ways the state has been responsive to the needs of families and adopted various FDC strategies. However, adoption is variable across agencies, and staff identified key areas of improvement including increasing opportunities for family peer support, providing transportation services for families, creating a family advisory board, and increasing opportunities for family-based treatment and parenting skills. Findings from this mixed methods assessment can inform strategic planning for the scale-up of FDC strategies across agencies in the state and can serve as a template for assessing strengths and weaknesses in the application of family engagement programs in systems nationally.

### Supplementary Information

**Supplementary Material 1. **

**Supplementary Material 2. **

## Data Availability

The datasets used and/or analyzed during the current study are available from the corresponding author on reasonable request.

## Abbreviations

FDC: Family-Driven Care
FGDM: Family Group Decision Making
FFT: Functional Family Therapy
JJ: Juvenile Justice
SAMHSA: Substance Abuse and Mental Health Services Administration

## References

[CR1] AACAP. (2003). AACAP/CWLA Statement on Use of Alcohol/Drugs, Screening/Assessment of Children in Foster Care. Retrieved from https://www.aacap.org/aacap/Policy_Statements/2003/Mental_Health_and_Use_of_Alcohol_and_Other_Drugs_Screening_and_Assesment_of_Children_in_Foster_Care.aspx

[CR2] Amani B, Milburn NG, Lopez S, Young-Brinn A, Castro L, Lee A, Bath E (2018). Families and the Juvenile Justice System: Considerations for Family-Based Interventions. Family & Community Health.

[CR3] The Annie E. Casey Foundation. (2014). Team Decision making: Engaging Families in Placement Decision. Retrieved February 21, 2024, from https://www.aecf.org/resources/team-decision-making.

[CR4] Arthur MW, Brown EC, Briney JS, Hawkins JD, Abbott RD, Catalano RF, Mueller MT (2015). Examination of substance use, risk factors, and protective factors on student academic test score performance. Journal of School Health.

[CR5] Arya N (2013). Family comes first: A workbook to transform the justice system by partnering with families.

[CR6] Becan JE, Fisher JH, Johnson ID, Bartkowski JP, Seaver R, Gardner SK, Blackwell L (2020). Improving substance use services for juvenile justice-involved youth: complexity of process improvement plans in a large scale multi-site study. Administration and Policy in Mental Health and Mental Health Services Research.

[CR7] Burke JD, Mulvey EP, Schubert CA (2015). Prevalence of mental health problems and service use among first-time juvenile offenders. Journal of Child and Family Studies.

[CR8] Burke JD, Mulvey EP, Schubert CA, Garbin SR (2014). The challenge and opportunity of parental involvement in juvenile justice services. Children Youth Serv Rev.

[CR9] Burke JD, Mulvey EP, Schubert CA, Garbin SR (2014). The Challenge and Opportunity of Parental Involvement in Juvenile Justice Services. Child Youth Serv Rev.

[CR10] Burrow-Sánchez JJ, Minami T, Hops H (2015). Cultural accommodation of group substance abuse treatment for Latino adolescents: Results of an RCT. Cultural Diversity Ethnic Minority Psychology.

[CR11] Burrow-Sanchez JJ, Wrona M (2012). Comparing culturally accommodated versus standard group CBT for Latino adolescents with substance use disorders: A pilot study. Cultural Diversity and Ethnic Minority Psychology.

[CR12] Cataldo, K., & Ford, K. (2010). Evaluation of the Colorado Integrated System of Care Family Advocacy Demonstration Programs for Mental Health Juvenile Justice Populations, Final Report. Bureau of Justice Assistance. Retrieved February 21, 2024, from https://www.ojp.gov/ncjrs/virtual-library/abstracts/evaluation-colorado-integrated-system-care-family-advocacy-0.

[CR13] Center for Juvenile Justice Reform. (2008). Juvenile justice professionals certificate program survey. Georgetown Public Policy Institute, Georgetown University, Washington, DC.

[CR14] Williams, J., Franks, R. P., & Dore, M. (2020). The Connecticut Juvenile Justice System: A Guide for youth and Families. Child Health and Development Institute of Connecticut, Inc. Retrieved 2024, from https://towyouth.newhaven.edu/wp-content/uploads/2020/09/CTJuvJusSystGuide.pdf.

[CR15] Coatsworth JD, Santisteban DA, McBride CK, Szapocznik J (2001). Brief strategic family therapy versus community control: Engagement, retention, and an exploration of the moderating role of adolescent symptom severity. Family Process.

[CR16] Crampton D (2003). Family group decision making in kent county, michigan: the family and community compact. Protecting Child.

[CR17] Dakof GA, Quille TJ, Tejeda MJ, Alberga LR, Bandstra E, Szapocznick J (2003). Enrolling and retaining mothers of substance-exposed infants in drug abuse treatment. J Consult Clin Psychol.

[CR18] DiClemente RJ, Davis TL, Swartzendruber A, Fasula AM, Boyce L, Gelaude D, Carry M (2014). Efficacy of an HIV/STI sexual risk-reduction intervention for African American adolescent girls in juvenile detention centers: a randomized controlled trial. Women Health.

[CR19] DMC Action Network. (2009). Defining "Cultural Competence": How Pierce County Is Engaging African-American Youth in Evidence-based Practices. Retrieved from http://bit.ly/1aoREdN.

[CR20] Dunst, C. J., & Trivette, C. M. (2009). Meta-analytic structural equation modeling of the influences of family-centered care on parent and Child Psychological Health. *International Journal of Pediatrics*, *2009*, 1–9. 10.1155/2009/576840. 10.1155/2009/576840 PMC2798088 20049341

[CR21] Dunst CJ, Trivette CM (2009). Meta-analytic structural equation modeling of the influences of family-centered care on parent and child psychological health. Int J Pediatrics.

[CR22] Dunst CJ, Trivette CM, Hamby DW (2007). Meta-analysis of family-centered helpgiving practices research. Ment Retard Dev Disabil Res Rev.

[CR23] Elkington KS, Bauermeister JA, Zimmerman MA (2010). Psychological distress, substance use, and HIV/STI risk behaviors among youth. Journal of youth and adolescence.

[CR24] Epstein, J. L. (2019). School, family, and community partnerships: Your handbook for action. Corwin, A SAGE Company.

[CR25] Pennsylvania’s Juvenile Justice System. (2012). A Family Guide to Pennsylvania’s Juvenile Justice System. Retrieved 2024, from http://www.pachiefprobationofficers.org/.

[CR26] Cohen, E., Ferguson, C., Berzin, S., Thomas, K., Lorentzen, B., & Dawson, W. (2004). California’s Title IV-E child welfare waiver demonstration project evaluation: Final report. University of California, Berkeley, School of Social Welfare, Child Welfare Research Center, Berkeley.

[CR27] Forde, S., & Schwartz, D. (2020). Georgia Department of Juvenile Justice YICPM Family Engagement Survey (pp. 1–7). GDJJ, Atlanta.

[CR28] Friedmann PD, Lemon SC, Stein MD (2001). Transportation and retention in outpatient drug abuse treatment programs. Journal of Substance Abuse Treatment.

[CR29] Georgia Department of Juvenile Justice. (2021). *Annual Report and Strategic Plan*. Retrieved 2024 from https://djj.georgia.gov/djj-publications

[CR30] Geurts EM, Boddy J, Noom MJ, Knorth EJ (2012). Family-centred residential care: The new reality?. Child & Family Social Work.

[CR31] Gonzales, Kane, Lopez-Howard, Devulapalli, Harper, & Fitts. (2018). DISPROPORTIONATE MINORITY CONTACT IN GEORGIA’S JUVENILE JUSTICE SYSTEM. Retrieved from https://cjcc.georgia.gov/grants/grant-subject-areas/juvenile-justice/state-advisory-group-sag/reddmc

[CR32] Haine-Schlagel R, Walsh NE (2015). A review of parent participation engagement in child and family mental health treatment. Clinical child and family psychology review.

[CR33] Hanes M (2012). In Focus: Disproportionate Minority Contact.

[CR34] Harris PA, Taylor R, Thielke R, Payne J, Gonzalez N, Conde JG (2009). Research electronic data capture (REDCap)—A metadata-driven methodology and workflow process for providing translational research informatics support. J Biomed Inform.

[CR35] Henggeler SW, Clingempeel WG, Brondino MJ, Pickrel SG (2002). Four-year follow-up of multisystemic therapy with substance-abusing and substance-dependent juvenile offenders. J Am Acad Child Adolesc Psychiatry.

[CR36] Hennink, M. M., Hutter, I., & Bailey, A. (2020). Qualitative research methods. Sage.

[CR37] Hoagwood KE, Cavaleri MA, Olin SS, Burns BJ, Slaton E, Gruttadaro D, Hughes R (2010). Family support in children’s mental health: A review and synthesis. Clinical Child and Family Psychology Rev.

[CR38] Hoeve M, McReynolds LS, Wasserman GA (2014). Service referral for juvenile justice youths: Associations with psychiatric disorder and recidivism. Adm Policy Ment Health.

[CR39] Hoeve M, McReynolds LS, Wasserman GA, McMillan C (2013). The influence of mental health disorders on severity of reoffending in juveniles. Criminal Justice Behavior.

[CR40] Hornberger S, Smith SL (2011). Family involvement in adolescent substance abuse treatment and recovery: What do we know? What lies ahead?. Child Youth Serv Rev.

[CR41] Horwitz SM, Chamberlain P, Landsverk J, Mullican C (2010). Improving the mental health of children in child welfare through the implementation of evidence-based parenting interventions. Administration and Policy in Mental Health and Mental Health Services Research.

[CR42] Hoytt, E. H., Schiraldi, V., Smith, B. V., & Ziedenberg, J. (2002). Reducing racial disparities in juvenile detention. Annie E. Casey Foundation, Baltimore. Retrieved on 2024 from https://www.aecf.org/resources/reducing-racial-disparities-in-juvenile-detention.

[CR43] IBM Corp. Released 2020. IBM SPSS Statistics for Windows, Version 27.0. IBM Corp, Armonk.

[CR44] Johnson, B., Abraham, M., Conway, J., Simmons, L., Edgman-Levitan, S., Sodomka, P., Schlucter, J., & Ford, D. (2008). Partnering with Patients and Families to Design and Patient and Family Centered Health Care System. Institute for Family-Centered Care. Retrieved 2024, from https://www.ipfcc.org/resources/PartneringwithPatientsandFamilies.pdf.

[CR45] National Institute of Corrections. (2012). Families unlocking futures: Solutions to the crisis in juvenile justice. Retrieved 2024, from https://nicic.gov/resources/nic-library/all-library-items/families-unlocking-futures-solutions-crisis-juvenile.

[CR46] Justice for Families. (2012). Families unlocking futures: Solutions to the crisis in juvenile justice. Retrieved from https://www.njjn.org/uploads/digital-library/Fam_Unlock_Future_EXEC_SUMNOEMBARGO.pdf

[CR47] Koroloff NM, Friesen BJ, Reilly L, Rinkin J (1996). The role of family members in systems of care.

[CR48] Kutash K, Duchnowski AJ, Green AL, Ferron JM (2011). Supporting parents who have youth with emotional disturbances through a parent-to-parent support program: A proof of concept study using random assignment. Administration and Policy in Mental Health and Mental Health Services Research.

[CR49] Latimer J (2001). A meta-analytic examination of youth delinquency, family treatment, and recidivism. Canadian Journal of Criminology.

[CR50] Leggatt M, Woodhead G (2016). Family peer support work in an early intervention youth mental health service. Early Intervention in Psychiatry.

[CR51] Lewis, R. A., & Judge, P. (2005). Family group conferencing: A realistic option for juvenile justice. American Humane FGDM Issues in Brief. Retrieved on 2024 from https://restorativejustice.org/rj-archive/family-group-conferencing-a-realistic-option-for-juvenile-justice/

[CR52] Liddle HA, Dakof GA, Henderson C, Rowe C (2011). Implementation outcomes of multidimensional family therapy-detention to community: a reintegration program for drug-using juvenile detainees. Int J Offender Ther Comp Criminol.

[CR53] Liddle HA, Rowe CL, Dakof GA, Henderson CE, Greenbaum PE (2009). Multidimensional family therapy for young adolescent substance abuse: twelve-month outcomes of a randomized controlled trial. Journal Consul Clin Psychol.

[CR54] Lindsey MA, Chambers K, Pohle C, Beall P, Lucksted A (2013). Understanding the behavioral determinants of mental health service use by urban, under-resourced Black youth: Adolescent and caregiver perspectives. Journal of Child and Family Study.

[CR55] Loyd AB, Hotton AL, Walden AL, Kendall AD, Emerson E, Donenberg GR (2019). Associations of ethnic/racial discrimination with internalizing symptoms and externalizing behaviors among juvenile justice-involved youth of color. Jouranl of Adolescence.

[CR56] Luckenbill, W. (2012). Innovation Brief: Strengthening the Role of Families in Juvenile Justice. Models for Change. Retrieved 2024, from https://modelsforchange.net/publications/352/.

[CR57] Marrett S (2017). Beyond rehabilitation: Constitutional violations associated with the isolation and discrimination of transgender youth in the juvenile justice system. BCL Rev..

[CR58] McCance-Katz, E. F. (2019). The national survey on drug use and health: 2017. Substance Abuse and Mental Health Services Administration. https://www.samhsa.gov/data/sites/default/files/nsduh-ppt-09–2018.pdf. Accessed 7 May

[CR59] McKay MM, McCadam K, Gonzales JJ (1996). Addressing the barriers to mental health services for inner city children and their caretakers. Community Mental Health Journal.

[CR60] McWayne C, Hampton V, Fantuzzo J, Cohen HL, Sekino Y (2004). A multivariate examination of parent involvement and the social and academic competencies of urban kindergarten children. Psychology in the Schools.

[CR61] Merikangas KR, He J-P, Brody D, Fisher PW, Bourdon K, Koretz DS (2010). Prevalence and treatment of mental disorders among US children in the 2001–2004 NHANES. Pediatrics.

[CR62] National Federation of Families for Childrens Mental Health. (2008). Working Definition of Family-Driven Care. Retrieved on 2024 from https://www.ffcmh.org/resources-familydriven.

[CR63] National Institute on Drug Abuse. (2000). Principles of drug addiction treatment: A research-based guide. National Institute on Drug Abuse, National Institutes of Health.

[CR64] National Center for Mental Health and Juvenile Justice. (2021). Family engagement in the Juvenile Justice System. Retrieved 2024, from https://www.ncsc.org/__data/assets/pdf_file/0023/80870/aecf-familyengagementframework-2021.pdf.

[CR65] Nellis, A., & Hooks, R. (2009). Back on Track: Supporting Youth Reentry from Out-of-Home Placement to the Community. Juvenile Justice and Delinquency Prevention Coalition. Retrieved 2024, from https://www.njjn.org/uploads/digital-library/resource_1397.pdf.

[CR66] Nolen S, McReynolds LS, DeComo RE, John R, Keating JM, Wasserman GA (2008). Lifetime suicide attempts in juvenile assessment center youth. Arch Suicide Res.

[CR67] Obrochta, C., Anthony, B., Armstrong, M., Kalil, J., Hust, J., & Kernan, J. (2011). Issue brief: Family-to-family peer support: Models and evaluation. ICF Macro, Outcomes Roundtable for Children and Families, Atlanta. Retrieved on 2024 from https://www.fredla.org/wp-content/uploads/2016/01/Issue-Brief_F2FPS.pdf.

[CR68] OJJDP. (2010). National Needs Assessment of Juvenile Justice Professionals: 2010. Office of Juvenile Justice and Delinquency Prevention, Office of Justice Programs, U.S. Department of Justice.

[CR69] OJJDP. (2013). Family Listening Sessions: Executive Summary. Office of Juvenile Justice and Delinquency Prevention, Office of Justice Programs, U.S. Department of Justice. Retrieved on 2024 from https://www.ojp.gov/library/publications/ojjdp-family-listening-sessions-executive-summary.

[CR70] Oldfield BJ, Harrison MA, Genao I, Greene AT, Pappas ME, Glover JG, Rosenthal MS (2019). Patient, family, and community advisory councils in health care and research: a systematic review. Journal of General Internal Medicine.

[CR71] Osher T, Penn M, Spencer S (2008). Partnerships with families for family-driven systems of care. The system of care handbook: Transforming mental health services for children, youth, and families.

[CR72] Osher TW, Osher D, Blau G, Gullotta TP, Blau GM (2008). Families matter. Family influences on childhood behavior and development: Evidence-based prevention and treatment approaches.

[CR73] Osher, T. W., Huff, B., Colombi, G. D., & and Gonsoulin, S. (2012). Family Guide to Getting Involved in Your Child’s Education at a Juvenile Justice Facility. Retrieved on 2024 from https://neglected-delinquent.ed.gov/sites/default/files/docs/NDTAC_FamilyGuide.pdf.

[CR74] Paik L (2017). Good parents, bad parents: Rethinking family involvement in juvenile justice. Theoretical Criminology.

[CR75] Pennell, J., Shapiro, C., & Spigner, C. (2011). Safety, fairness, stability: Repositioning juvenile justice and child welfare to engage families and communities. Retrieved from https://juvenilecouncil.ojp.gov/sites/g/files/xyckuh301/files/media/document/center_for_juvenile_justice_reform_paper_web.pdf.

[CR76] Pennell J, Burford G (2000). Family group decision making: protecting children and women. Child welfare.

[CR77] Pennsylvania’s Family Group Decision Making (FGDM) Leadership Team. (2009). Pennsylvania Family Group Decision Making Toolkit: A Resource to Guide and Support Best Practice Implementation. Retrieved from https://nccwe.org/toolkits/family-engagement/shared_planning_decision_making.htm.

[CR78] Peterson-Badali M, Broeking J (2010). Parents’ involvement in the youth justice system: Rhetoric and reality. Canadian Journal of Criminology and Criminal Justice.

[CR79] Piper, K. N., Pankow, J., & Wood, J. D. (2023). Juvenile probation staff perceptions of engaging families in substance use services. Family Relations, 1–24. 10.1111/fare.1297. 10.1111/fare.12974 PMC11175584 38881821

[CR80] Poteat VP, Scheer JR, Chong ES (2016). Sexual orientation-based disparities in school and juvenile justice discipline: A multiple group comparison of contributing factors. Journal of Educcational Psychology.

[CR81] Prendergast M, Welsh WN, Stein L, Lehman W, Melnick G, Warda U, Abdel-Salam S (2017). Influence of organizational characteristics on success in implementing process improvement goals in correctional treatment settings. Journal of Behavioral Health Services and Research.

[CR82] Prison Policy Initiative. (2019). Youth confinement: The whole pie. Youth confinement: The whole pie 2019. In: Author. Retrieved from https://www.prisonpolicy.org/reports/youth2019.html.

[CR83] Purdy, F. (2010). The core competencies for parent support providers. National Federation of Families for Children's Mental Health, Rockville. Retrieved from https://www.pacesconnection.com/fileSendAction/fcType/0/fcOid/476162897103902931/filePointer/476162897133942694/fodoid/476162897133942688/FFCMH%203-Competencies-Brief.pdf.

[CR84] Puzzanchera, C. (2021). Juvenile Justice Statistics National Report Series Bulletin: Juvenile Arrests, 2019. Office of Juvenile Justice and Delinquency Prevention. Retrieved from https://ojjdp.ojp.gov/publications/juvenile-arrests-2019.pdf.

[CR85] Robbins, V., Johnston, J., Barnett, H., Hobstetter, W., Kutash, K., Duchnowski, A., & Annis, S. (2008). Parent to parent: A synthesis of the emerging literature. Tampa, FL: University of South Florida, The Louis de la Parte Florida Mental Health Institute, Department of Child & Family Studies. Retrieved from http://cfs.cbcs.usf.edu/_docs/publications/parent_to_parent.pdf.

[CR86] Robertson AA, Hiller M, Dembo R, Dennis M, Scott C, Henry BF, Elkington KS (2019). National survey of juvenile community supervision agency practices and caregiver involvement in behavioral health treatment. J Child Family Stud.

[CR87] Sales JM, Wasserman G, Elkington KS, Lehman W, Gardner S, McReynolds L, Knudsen H (2018). Perceived importance of substance use prevention in juvenile justice: a multi-level analysis. Health Jus.

[CR88] Santisteban DA, Szapocznik J, Perez-Vidal A, Kurtines WM, Murray EJ, LaPerriere A (1996). Efficacy of intervention for engaging youth and families into treatment and some variables that may contribute to differential effectiveness. J Family Psychol.

[CR89] Schubert CA, Mulvey EP, Glasheen C (2011). Influence of mental health and substance use problems and criminogenic risk on outcomes in serious juvenile offenders. J Ame Acad Child Adolesc Psychiatry.

[CR90] Shanahan, R., & Agudelo, S. V. (2011). Families as Partners: Supporting Incarcerated Youth in Ohio. Vera Institute of Justice, New York. Retrieved from https://www.vera.org/downloads/publications/families-as-partners.pdf.

[CR91] Shanahan, R., & Agudelo, S. V. (2012). Families as Partners: Supporting Incarcerated Youth in Ohio. Retrieved from https://storage.googleapis.com/vera-web-assets/downloads/Publications/families-as-partners-supporting-incarcerated-youth-in-ohio/legacy_downloads/families-as-partners.pdf

[CR92] Shanahan R, diZerega M (2016). Identifying, engaging, and empowering families: A charge for juvenile justice agencies: Center for Juvenile Justice Reform.

[CR93] Sheets J, Wittenstrom K, Fong R, James J, Tecci M, Baumann DJ, Rodriguez C (2009). Evidence-based practice in family group decision-making for Anglo, African American and Hispanic families. Children Youth Serv Rev.

[CR94] Slavet, J. D., Stein, L. A., Klein, J. L., Colby, S. M., Barnett, N. P., & Monti, P. M. (2005). Piloting the Family Check-Up With Incarcerated Adolescents and Their Parents. *Psychological Services, 2*(2), 123. Retrieved from https://www.ncbi.nlm.nih.gov/pmc/articles/PMC2743104/pdf/nihms135514.pdf. 10.1037/1541-1559.2.2.123 PMC2743104 19756250

[CR95] Smelstor, L. (2000). A Handbook for Parents of Children Committed to the Massachusetts Department of Youth Services. Retrieved from Boston, Massachusetts: https://www.njjn.org/uploads/digital-library/resource_153.pdf.

[CR96] Spencer SA, Blau GM, Mallery CJ (2010). Family-driven care in America: More than a good idea. J Can Acad Child Adolesc Psychiatry.

[CR97] Spinney E, Cohen M, Feyerherm W, Stephenson R, Yeide M, Shreve T (2018). Disproportionate minority contact in the US juvenile justice system: A review of the DMC literature, 2001–2014, Part I. J Crime Jus.

[CR98] Spoth R, Redmond C (1993). Study of participation barriers in family-focused prevention: Research issues and preliminary results. Int Q Community Health Educ.

[CR99] Spoth R, Redmond C (1995). Parent motivation to enroll in parenting skills programs: A model of family context and health belief predictors. J Fam Psychol.

[CR100] Spoth R, Redmond C, Shin C (2000). Modeling factors influencing enrollment in family-focused preventive intervention research. Prev Sci.

[CR101] Syed ST, Gerber BS, Sharp LK (2013). Traveling towards disease: transportation barriers to health care access. J Community Health.

[CR102] Szapocznik J, Perez-Vidal A, Brickman AL, Foote FH, Santisteban D, Hervis O, Kurtines WM (1988). Engaging adolescent drug abusers and their families in treatment: A strategic structural systems approach. J Consult Clin Psychol.

[CR103] Taxman, F. S., Henderson, C. E., & Belenko, S. (2009). Organizational context, systems change, and adopting treatment delivery systems in the criminal justice system. *Drug and Alcohol Dependence*, *103*, S1-S6. 10.1016/j.drugalcdep.2009.03.003 19423241

[CR104] Teplin LA, Abram KM, McClelland GM, Dulcan MK, Mericle AA (2002). Psychiatric disorders in youth in juvenile detention. Archives of General Psychiatry.

[CR105] Teplin LA, Mericle AA, McClelland GM, Abram KM (2003). HIV and AIDS risk behaviors in juvenile detainees: implications for public health policy. Am J Public Health.

[CR106] Tolou-Shams M, Stewart A, Fasciano J, Brown LK (2010). A review of HIV prevention interventions for juvenile offenders. J Pediatric Psychol.

[CR107] Trupin EJ, Kerns SE, Walker SC, DeRobertis MT, Stewart DG (2011). Family integrated transitions: A promising program for juvenile offenders with co-occurring disorders. Journal of Child & Adolescent Substance Abuse.

[CR108] Head Start. (2024). Parent, family, and community engagement (PFCE) framework. Head Start ECLKC. https://eclkc.ohs.acf.hhs.gov/school-readiness/article/parent-family-community-engagement-pfce-framework.

[CR109] Van der Pol, T., Hoeve, M., Noom, M., Stams, G., Doreleijers, T., Van Domburgh, L., & Vermeiren, R. (2017). The effectiveness of multidimensional family therapy (MDFT) in treating substance abusing adolescents with comorbid behavior problems: a meta-analysis. J Child Adolesc Psychiatry. 10.1111/jcpp.12685 28121012

[CR110] Van der Put CE, Creemers HE, Hoeve M (2014). Differences between juvenile offenders with and without substance use problems in the prevalence and impact of risk and protective factors for criminal recidivism. Drug Alcohol Depend.

[CR111] Vera Institute of Justice. (2014). Family Engagement in the Juvenile Justice System: Juvenile Justice Fact Sheet 5. Retrieved from New York, NY: https://www.vera.org/downloads/Publications/family-engagement-in-the-juvenile-justice-system/legacy_downloads/family-engagement-juvenile-justice.pdf

[CR112] VERBI Software. (2022). MAXQDA 2022. Berlin, Germany: VERBI Software. Retrieved from maxqda.com

[CR113] Walker, S., Pullmann, M., Trupin, E., Hansen, J., & Ague, S. (2011). A Guidebook for Implementing Juvenile Justice 101. Retrieved from

[CR114] Walker SC, Bishop AS, Pullmann MD, Bauer G (2015). A research framework for understanding the practical impact of family involvement in the juvenile justice system: the juvenile justice family involvement model. Am J Community Psychol.

[CR115] Wasserman GA, McReynolds LS (2006). Suicide risk at juvenile justice intake. Suicide and Life-Threatening Behavior.

[CR116] Wasserman GA, McReynolds LS, Schwalbe CS, Keating JM, Jones SA (2010). Psychiatric disorder, comorbidity, and suicidal behavior in juvenile justice youth. Criminal Justice and Behavior.

[CR117] Wasserman GA, McReynolds LS, Taxman FS, Belenko S, Elkington KS, Robertson AA, Dembo R (2021). The missing link (age): Multilevel contributors to service uptake failure among youths on community justice supervision. Psychiatric Services.

[CR118] Weigensberg EC, Barth RP, Guo S (2009). Family group decision making: A propensity score analysis to evaluate child and family services at baseline and after 36-months. Children and youth services review.

[CR119] Welti, Wilkins, & Malm. (2021). Evaluation of Los Angeles County’s Upfront Family Finding Program | Phase 2. Retrieved from https://www.childtrends.org/publications/evaluation-of-los-angeles-countys-upfront-family-finding-program-phase-2

[CR120] Williamson E, Gray A (2011). New roles for families in child welfare: Strategies for expanding family involvement beyond the case level. Children and youth services review.

[CR121] Willison, J. B., Brooks L., Salas M., Dank M., Denver M., Gitlow E., Roman J. K., Butts J. A. (2010). Reforming Juvenile Justice Systems: Beyond Treatment. A Reclaiming Futures National Evaluation Report. Reclaiming Futures National Program Office, Portland State University, Portland. Retrieved from https://www.urban.org/sites/default/files/publication/28026/1001366-Reforming-Juvenile-Justice-Systems.PDF.

[CR122] Woolfenden, SR, Williams, K, Peat, JK. (2002). Family and parenting interventions for conduct disorder and delinquency: a meta-analysis of randomised controlled trials. Archives of disease in childhood, 86(4), 251–256. Retrieved from https://www.ncbi.nlm.nih.gov/pmc/articles/PMC1719168/pdf/v086p00251.pdf 10.1136/adc.86.4.251 PMC1719168 11919097

[CR123] Zoom Video Communications Inc. (2023). Zoom Version: 5.13.3. Retrieved from https://zoom.us/

