# Supplementary material for: A mixed methods evaluation of family-driven care implementation in juvenile justice agencies in Georgia
Source: Health Justice. 2024 Feb 26;12:8. doi: 10.1186/s40352-024-00261-0 (PMC10895769; doi:10.1186/s40352-024-00261-0)
Supplement: Supplementary file 1 — Supplementary Material 1. [file 40352_2024_261_MOESM1_ESM.docx]

Additional File 1. Juvenile Justice Staff Interview Guide

*Opening Questions*

1. What is your job title and your job responsibilities?
2. Can you share a little bit about the office you work at and describe the work that your agency does?

*Family Needs*

1. Can you describe the most common behavioral health needs of families that you work with?
   1. Mental health?
   2. Substance use?
   3. Sexual/reproductive health?
2. What services are available to support justice-involved youth and families?
3. What services are needed to support justice-involved families?
4. Currently, what role does your agency play in addressing the needs of justice-involved families?
   1. In your opinion, what role should juvenile justice systems be taking to address family needs, if any?

*Family Engagement Climate and Attitudes*

1. When I say family engagement, what does this mean to you?
2. Can you describe the family engagement practices that you or your agency utilize, if any?
   1. Probe if needed:
      - How does your agency involve parents/guardians in decisions related to youths’ service or treatment planning, if at all?
      - What other supports or programs (e.g., family support groups, parenting classes, transportation/childcare, etc.) do you currently provide to families at your agency, if any?
      - How does your agency identify and meet the needs of families from diverse racial/ethnic backgrounds?
      - What techniques are used at your agency to ensure that families fully understand all the processes of the juvenile justice system?
      - How are parents given opportunities to provide feedback to staff in your agency about what is and is not working?
3. How has the COVID-19 pandemic impacted how you work with families, if at all?
4. Overall, how would you describe the relationship between staff and families at your agency?
   1. How do staff at your agency feel about working with families?
   2. What challenges do staff face when interacting with families at your agency?
   3. What makes interactions with families go more smoothly?
5. At your agency, how much of a priority is family engagement?
   1. Why do you think that is?
6. To what extent do DJJ leadership and leaders at your agency support family engagement?
   1. Are there people in your organization who champion family engagement (i.e., go above and beyond what might be expected)?
      - Is there anyone who is leading change efforts related to family engagement?
      - Can you tell me a little about why you think they champion family engagement?
7. How does your agency collaborate with external partners/organizations to support youth and families?

*Training and Resources*

1. How have staff at your agency been trained on family engagement practices, if at all?
   1. What training would you and staff in your agency need in this area?
   2. What types of trainings work well at your agency?
2. What is your organization’s capacity to expand family engagement practices and services?
   1. What are the obstacles to expanding family engagement practices in your agency?
   2. What support and resources would you need to enhance family engagement at your agency?

*Closing Questions*

1. Based on what we have discussed today, what improvements to family engagement would you like to see implemented in the next year within your agency?
2. What additional recommendations do you have for enhancing family engagement in juvenile justice settings?
3. Is there anything else you think that we should know about family engagement that we have not yet covered? *Thank you so much for your time today.*
