# Supplementary material for: A mixed methods evaluation of family-driven care implementation in juvenile justice agencies in Georgia
Source: Health Justice. 2024 Feb 26;12:8. doi: 10.1186/s40352-024-00261-0 (PMC10895769; doi:10.1186/s40352-024-00261-0)
Supplement: Supplementary file 2 — Supplementary Material 2. [file 40352_2024_261_MOESM2_ESM.docx]

Additional File 2. Mixed Methods Data Comparison Matrix

| **Domain** | **Strategies** | **Findings from Survey Ratings** | **Findings from Bivariate Analyses** | **Findings from Qualitative Data** | **Example Quotes** |
| --- | --- | --- | --- | --- | --- |
| **Identifying and Involving Family Voices** | Individuals at my agency systematically identify members of each youth’s family unit. | 77% of participants either agreed or strongly agreed that this strategy was implemented in their agencies. | There are no differences in implementation of this strategy across organizational and staff characteristics. | Parents and guardians are identified and involved from the very beginning of youth's encounter with the justice system. When parents/guardians are unable to participate, JJ staff work with the child to identify and involve other members of the family unit (such as siblings, grandparents, and other extended family members) that can serve as advocates for the youth throughout their involvement with the system. Although the department tries to involve a family advocate in all processes and decisions, not every youth has a family member that is willing and able to participate. Interviewees noted that some families are unable to engage in JJ processes (due to competing demands, financial insecurity, or other logistical barriers like transportation), or they are unwilling to engage with JJ staff (due to mistrust and strained interpersonal relationships with staff). Participants believed that the level of family involvement and “buy-in” to the service provision process are directly linked to youth youth’s success | "There’s not too much that we can do without the parent, because they’re children. So even from the beginning of the – their involvement in the justice system, the parent has to show up in court. The parent has to have some accountability, even in the order of probation that is written. The parent has to sign it as well because if the parent – if the child let’s say has to report to the probation office, the parent has to commit to bringing that child to probation. If the child is referred to mental health services, substance abuse treatment or whatever it is, one time a week, or family therapy, the parent has to bring that child to where they need to be and also participate in that family therapy. So there are certain tenants that are there just because they’re children, that the parents are going to be called to do, because they’re responsible for their children.” (ID: #2, Leadership, Reentry Services)  "When you say family engagement, it means involving anybody that touches the youth, that they feel important in their life. And so that’s not necessarily confined to just a biological parent – But it’s guardians or somebody that – or extended family members as well as important people to the family, that they regard as family." (ID: #15, Leadership, Reentry Services)  “we definitely do our due diligence in trying to ensure that we allow the families to participate as well as provide the option that if they aren’t able to participate, that they can have someone stand in on their behalf that they feel comfortable.” (ID: #10, Staff, Reentry Services)   "If we have the parent buy in, their support, I’ve seen it where the parent from the time we do their intake appointment, all the way through the whole process that we’re supervising that case, their buy in and listening and being able to have that open line of communication, to make sure that the kid gets everything they need to get them back on track is key and when we don’t have that parent buy in, it can be a barrier sometimes....If we don’t have the buy in from the parent, that’s going to be a barrier to getting kids to groups or getting the kids to counseling appointments, getting the kid whatever resources they need." (ID: #1, Leadership, Community Services) |
|  | In my agency, all youth have a biological, adoptive, foster, or surrogate family voice advocating on their behalf. | 52% of participants either agreed or strongly agreed that this strategy was implemented in their agencies. | There are no differences in implementation of this strategy across organizational and staff characteristics. |  |  |
| **Informing Families** | In my agency, families are given accurate, understandable, and complete information necessary to set goals and make informed decisions about the right services and supports for their children. | 88% of participants either agreed or strongly agreed that this strategy was implemented in their agencies. | There are no differences in implementation of this strategy across organizational and staff characteristics. | Participants discussed how families are informed at all steps in the process, and there are policies in place to ensure they are informed in a timely, transparent, and accurate manner, starting with an initial intake and orientation meeting. Staff expressed that keeping the families up to date with any new information is a priority within the department. In addition to face-to-face meetings, staff utilize various modes of communication including email, text, call, or videoconferencing to keep the family informed as engaged. They also provided informational brochures on JJ system procedures as well as informational materials related to resources and services available in their community. Participants voiced that open, transparent, consistent communication builds a better relationship with family members. | “They’re informed about if something happens within a certain timeframe and policies are in place to support that, so it’s not like an individual’s decision about how to make that happen. There are policies that are out there.” (ID: #16, Staff, Reentry Services)   “I mean, you’re engaging family from the time that this kid enters into the justice system, whether they’re in the community or they are in detention, I mean, because you have to communicate with the parents to understand what they expect to happen throughout this process, to help them understand what the next steps are, see what the expectations are, the things that they feel that the individual needs.” (ID: #5, Staff, Community Services)   “One of the measures that we use is we have a required standards of contact [with families] – depending on the child’s arrest level or their level of supervision and based on that standards of contact, we have an intake appointment with the families and explain to them what the expectations are, to ensure that we’re all on the same page in regards to how often the child needs to be seen, how often do we need to see the parent, how many phone calls we need to make a phone, collaborations and collaborative meetings. So that’s one of the measures that we use agency wide in regards to how we communicate with the families, just having that initial intake appointment for them to understand that.” (ID: #1, Leadership, Community Services)   "And there are some things that we do with the schools in terms of family – family events that’s through the education system. Just making sure that those services that we are providing the youth, the family is fully aware of them and participating as much as they want to or can in that process.” (ID: #15, Leadership, Reentry Services) |
|  | Individuals in my agency educate family members on the procedures and policies of the juvenile justice system. | 76% of participants either agreed or strongly agreed that this strategy was implemented in their agencies. | There are no differences in implementation of this strategy across organizational and staff characteristics. |  |  |
| **Collaborative Decision Making and Care Planning** | In my agency, families and staff embrace the concept of sharing decision-making and responsibility for outcomes. | 72% of participants either agreed or strongly agreed that this strategy was implemented in their agencies. | There are no differences in implementation of this strategy across organizational and staff characteristics. | JJ staff partner with families involved in the system to make collaborative care decisions. This ensures that families' opinions and preferences are integrated into their child's care plan. This is a priority for staff to ensure families are ready for their child to return home when the time comes. Working alongside the family enables the youth to remain a part of the family unit while they may be away from home. The Youth-Centered Re-entry Team (YCRT) meeting is held for families to participate in decision making for the youth’s service planning and encourages collaboration between family and staff. Overall, staff communicated that the family’s participation in the youth’s care facilitates a more successful outcome for youth and their family. | “Because if the family’s not involved, how can you welcome their child back home? So, you’ve got to have them involved in the consultation and the discussion of the care and the discussion of the education and discussion of the medication. They’ve got to be involved. If you continue to involve them, then they’re more receptive to their child coming back home who they just- you know, they don’t- somebody else has been raising them for that timeframe and to keep them a part of the conversation, then that has helped out with them being ready to accept the kid coming back home.” (ID: #16, Staff, Reentry Services)  “Also with our reentry framework, the parent or the family is very involved in that reentry planning phase, so that it is a collaborative effort. We don’t do things outside of the family knowing, you know, the youth ultimately is returning to the family, so they need to be a part of that process.” (ID: #12, Leadership, Reentry Services)   “I think we play a big role in making sure that we are not only addressing the youth themselves that are involved in the criminal justice system, but also engaging the family to make sure that it is a unit, a group project. We definitely want that parental feedback and support, because those are the things that drive the youth forward and ultimately can enable to the family to be better.” (ID: #5, Staff, Community Services)   “Family engagement means allow the families to have a voice and a say in what is essential for their success and for the youth’s transition back to home and to various practices that are occurring within the agency” (ID: #10, Staff, Reentry Services) |
|  | In my agency, staff advocate for the needs and preferences of families and youth. | 82% of participants either agreed or strongly agreed that this strategy was implemented in their agencies. | There are no differences in implementation of this strategy across organizational and staff characteristics. |  |  |
|  | Individuals in my agency involve families in treatment planning (e.g., family-group decision making or family-group conferencing). | 81% of participants either agreed or strongly agreed that this strategy was implemented in their agencies. | This strategy is more likely to be implemented in community supervision settings (compared to detention settings) and most likely to be implemented by line staff and case managers/reentry planners. |  |  |
| **Organizational Decision Making** | Individuals in my agency encourage families to provide formal feedback on system processes (e.g., through family surveys and/or family town halls). | 69% of participants either agreed or strongly agreed that this strategy was implemented in their agencies. | There are no differences in implementation of this strategy across organizational and staff characteristics. | Interviewees discussed how families are invited to provide feedback on organizational procedures through several methods, including through the Ombudsman office, through family surveys, and during family roundtable sessions (called The Chat). Staff also hoped that families feel comfortable approaching them with any concerns, and many line staff check-in with families regularly through phone calls or collaborative care meetings An advisory board is not currently utilized within JJ, however, staff discussed the hope that one will be established in the future as family's opinions are essential to the organization. | “We’ve been working on a parent advisory committee. I would love to see us have one of those where we have parents involved around the table to tell us how we can better serve them.” (ID: #2, Leadership, Reentry Services)   “They have the opportunity to voice their opinion in the YCRT team meeting, that is an open forum and discussion. … And so if there are concerns on behalf of the family, they have the ability to bring those questions or concerns to the table, and they can be discussed at that time.” (ID: #12, Leadership, Reentry Services)   “We continue to connect them to services, we have the Chat, which is a family engagement call that allows families to come and hear about our resources, as well as room for them to bring up any concerns that they may have.” (ID: #10, Staff, Reentry Services)   “They certainly have opportunity to give feedback to me. It’s not a thing of them having to figure out how do I do this. I go after it. I will ask them, what’s working, what’s not, what do we need to change, how can we adapt?” (ID: #4, Staff, Community Services) |
|  | Individuals in my agency Invite family representatives to serve on advisory boards or policy-making committees. | 35% of participants either agreed or strongly agreed that this strategy was implemented in their agencies. | This strategy is more likely to be implemented in detention settings (compared to community supervision settings). |  |  |
| **Family Peer Support** | In my agency, families are provided opportunities to engage in peer support activities and connect with other parents/guardians of justice-involved youth. | 52% of participants either agreed or strongly agreed that this strategy was implemented in their agencies. | There are no differences in implementation of this strategy across organizational and staff characteristics. | The department holds various events and meetings which provide an avenue for families to connect with one another. The Chat and the Family Cafe are held regularly to encourage connection among families. Additionally, there was a parent counseling group in the past at one of the facilities. Staff are hopeful to get this group back up and running to provide another opportunity for family connection and peer support. | “Family café, they do – it’s parenting skills, but it also allows the family to have a platform to connect with other parents or guardians who are in the same position as them, so some – it allows an area of support.” (ID: #10, Staff, Reentry Services)   “From time to time do have different support groups, parents or family members identify things that are concerning, we make sure we follow up and try to address those concerns. It’s not just limited to parents who have youth still in secure confinement, but anybody that wants to come into that chat or anybody that might have – have a youth that’s been involved at some point and through those chats, we also provide classes and different presentations for the participants. And each month we might focus on something different. We’ve had cooking classes where they would cook and got certain foods and utensils at the end of the course.” (ID: #15, Leadership, Reentry Services)  “We had a mental health counselor who came in on weekends during visitation and those parents who wished to participate could stay for a parent counseling group. And, yeah, the parents loved it, you know, and it was kind of part counseling, part planning and part peer support. And the parents just loved it. I would love to be able to offer something like that at all of the 25 facilities, you know, and have staff who are dedicated to coming in on the weekends when it’s convenient for the parents and doing that.” (ID: #7, Leadership, Behavioral Health) |
|  | Individuals in my agency provide opportunities for family members to participate in support groups. | 57% of participants either agreed or strongly agreed that this strategy was implemented in their agencies. | There are no differences in implementation of this strategy across organizational and staff characteristics. |  |  |
| **Logistical Support** | Individuals in my agency provide flexible scheduling to accommodate families. | 78% of participants either agreed or strongly agreed that this strategy was implemented in their agencies. | This strategy is more likely to be implemented in community supervision settings (compared to detention settings), and by case managers, line staff, and admin. | Logistical support is provided by staff at JJ. During Covid-19, staff pivoted to virtual meetings with families and ensured they had access to communicate with their children through virtual means as in-person visitation was not allowed. This adaptation increased accessibility for families to participate in their youth's service planning and communicate without a potential commute to the facility. Staff also communicated that the necessity of providing transportation, gas cards, or other compensation to encourage families to visit the facilities. In addition to utilizing virtual communication, participants also discussed other avenues that they help families overcome logistical challenges, such as through helping them schedule and access referral appointments | “And so if I’m asking you to come participate in a family advisory committee, you may have three little ones at home, so I may need to help provide child care so that you can be available. Or if you come to this meeting and you have a family of five at home, who is preparing dinner? So I may need to give you a gift card or something to have pizza brought in, or take dinner home to the family once we’re done with the meeting. We’re not paying families to partner with us, but we understand that families have specific needs and if we really want to engage with them and glean valuable information from them, then there needs to be something on our side that says we know that this may present a hardship and here’s something to help with that.” (ID: #12, Leadership, Reentry Services)   “So we did a lot of different things, we learned a lot of different things. We learned how to utilize technology quite a bit more. And so we learned that that’s one way to allow one of our youths who parents may not be able to necessarily visit with them as much but virtually, you know, if they could – they could communicate and have some time with their youth.” (ID: #2, Leadership, Reentry Services)   “And I still feel in person is very effective, but one thing we did learn from COVID-19, you can get a lot more participation and a broader scope in terms of the participants from a virtual platform, because people can actually access those services from wherever they are.” (ID: #15, Leadership, Reentry Services)   “But another major challenge I think is helping not just the youth, but recognizing that there’s a family situation that you need to assist them with and like I said, transportation is one of those things. Making sure that they have the things that are needed as it relates to being able to engage in those services, like proper identification. We have programs for that. Making sure that they’re not just told where their appointments or referrals are, but helping them make those appointments and facilitating the process to make sure they’re able to get there.” (ID: #15, Leadership, Reentry Services)  “Well, I think we’re continuing to expand, particularly with the re-entry initiative, because every week we’re getting more and more partners in the community that are going to help the kids and we’re trying to engage – like we have a clothing closet now too that the parents can go and get clothing for free. We’ve got gas cards.” (ID: #6, Leadership, Behavioral Health) |
|  | Individuals in my agency assist families with transportation needs. | 27% of participants either agreed or strongly agreed that this strategy was implemented in their agencies. | There are no differences in implementation of this strategy across organizational and staff characteristics. |  |  |
|  | Individuals in my agency assist families with childcare needs. | 18% of participants either agreed or strongly agreed that this strategy was implemented in their agencies. | There are no differences in implementation of this strategy across organizational and staff characteristics. |  |  |
|  | In my agency, staff work to remove family barriers to engagement and participation. | 71% of participants either agreed or strongly agreed that this strategy was implemented in their agencies. | There are no differences in implementation of this strategy across organizational and staff characteristics. |  |  |
| **Family Diversity and Inclusion** | In my agency staff embrace, value, and celebrate the diverse cultures of their youth and families. | 77% of participants either agreed or strongly agreed that this strategy was implemented in their agencies. | This strategy is more likely to be implemented in community supervision settings (compared to detention settings). | Staff are continually trained on diversity, equity, and inclusion principles. Additionally, DJJ focuses on having a diverse staff and mentors to ensure adequate representation among their staff. In addition to training and promoting workforce diversity, the department has mechanisms to monitor inequities in the system and respond to family grievances: | “We definitely train our staff in terms of cultural sensitivity, cultural implicit bias training, things of that nature, as we work with our youth and our families.” (ID: #2, Leadership, Reentry Services)   “By making sure our team is racially diverse, number one. And making sure that we understand that we have to be sensitive and aware of racial diversity. But I think when you’ve got people that come from diverse communities and whatnot, they have a more personal understanding about what that really means.” (ID: #15, Leadership, Reentry Services)  “We also are trying to look at making sure that we’re not creating any disparities in our health services or any of the other services that we do because we have a quality assurance monitoring program where we look at health services. … And we do a lot around the facility, there’s a grievance system for young people to utilize. … So there are several mechanisms that both the youth can utilize and the parent can utilize when they feel like they’re not being treated fairly.” (ID: #6, Leadership, Behavioral Health) |
|  | In my agency, staff continually advance their own cultural and linguistic responsiveness, so that the needs of all families are appropriately addressed. | 64% of participants either agreed or strongly agreed that this strategy was implemented in their agencies. | There are no differences in implementation of this strategy across organizational and staff characteristics. |  |  |
|  | Individuals in my agency, receive formal training on diversity and inclusion (e.g., cultural humility training, racial sensitivity training, and/or unconscious bias training). | 64% of participants either agreed or strongly agreed that this strategy was implemented in their agencies. | This strategy is more likely to be implemented in community supervision settings (compared to detention settings). |  |  |
| **Family Health and Functioning** | Individuals in my agency provide parenting skills programs. | 40% of participants either agreed or strongly agreed that this strategy was implemented in their agencies. | There are no differences in implementation of this strategy across organizational and staff characteristics. | Staff indicated that they offered some parenting skills programs, which they are trying to expand and offer to more parents. Their parenting skills program (called Family Café) utilizes an evidence-base curriculum, which involves a series of presentations and workshops for families to learn skills that can be applied when their children return home.  Different services are referred out to providers outside of the DJJ. Staff discussed the use of wraparound providers to deliver therapeutic services to children and their families. They also emphasized the importance of connecting families to behavioral health services, whether they are provided in-house or referred out. | “We’re relaunching our second session of the family café, kind of like parent university...it’s parenting skills, but it also allows the family to have a platform to connect with other parents or guardians who are in the same position as them. It allows an area of support.” (ID: #10, Staff, Reentry Services)   “We do offer some programs and supports through initiatives like the Family Café that we offer, which uses an evidence-based curriculum and teaches Active Parenting. We offer a nurturing parenting curriculum for our youth and families.” (ID: #12, Leadership, Reentry Services)   “We have what’s called The Chat, where it’s a five-week series in the evenings with families, preparing them for those youth that are coming back home where they’re taught parenting skills and have conversations with parents.” (ID: #16, Staff, Reentry Services)  “Typically we use services that are kind of wrap around services. So we’ll have like one vendor that will be able to address the individual counseling, the family therapy, as well as like substance abuse and other things that the individual may need, as well as stuff like MST, which is the multisystemic therapy. And they also work with the family as well as the individual youth to teach them coping mechanisms as well as life skills and things like that. We also utilize mentoring, in which they kind of engage in the family as well, to kind of make sure that the individuals are moving towards their more prosocial activity aspect as well as like I said, those life skills that are really important. But that’s pretty much it. Just different wraparound providers.” (ID: #5, Staff, Community Supervision)   “It is left up to them to participate, but when you talk about those mental health requirements, there may be things for multisystemic therapy, MST, FFT, the functional family therapy requirements, and that’s because to- that the relationship may have been impacted because the youth is confined, so how do we prepare that family.” (ID: #16, Staff, Reentry Services) |
|  | Individuals in my agency refer to parenting skills programs. | 51% of participants either agreed or strongly agreed that this strategy was implemented in their agencies. | This strategy is more likely to be implemented in community supervision settings (compared to detention settings). |  |  |
|  | Individuals in my agency provide family-based mental health services (e.g., family counseling, therapy, mental health treatment). | 58% of participants either agreed or strongly agreed that this strategy was implemented in their agencies. | There are no differences in implementation of this strategy across organizational and staff characteristics. |  |  |
|  | Individuals in my agency refer to family-based mental health services (e.g., family counseling, therapy, mental health treatment). | 76% of participants either agreed or strongly agreed that this strategy was implemented in their agencies. | This strategy is more likely to be implemented in community supervision settings (compared to detention settings), and most likely to be implemented by case managers, line staff, and admin. |  |  |
|  | Individuals in my agency provide family-based substance use services (e.g., family substance use prevention or treatment). | 44% of participants either agreed or strongly agreed that this strategy was implemented in their agencies. | There are no differences in implementation of this strategy across organizational and staff characteristics. |  |  |
|  | Individuals in my agency refer to family-based substance use services (e.g., family substance use prevention or treatment). | 62% of participants either agreed or strongly agreed that this strategy was implemented in their agencies. | This strategy is more likely to be implemented in community supervision settings (compared to detention settings), and most likely to be implemented by case managers and line staff. |  |  |
|  | Individuals in my agency provide family-based HIV/STI services (e.g., family-based HIV/STI prevention or treatment). | 23% of participants either agreed or strongly agreed that this strategy was implemented in their agencies. | There are no differences in implementation of this strategy across organizational and staff characteristics. |  |  |
|  | Individuals in my agency refer to family-based HIV/STI services (e.g., family-based HIV/STI prevention or treatment). | 32% of participants either agreed or strongly agreed that this strategy was implemented in their agencies. | There are no differences in implementation of this strategy across organizational and staff characteristics. |  |  |
